# Electrospun hybrid nanofibers: Fabrication, characterization, and biomedical applications

**DOI:** 10.3389/fbioe.2022.986975

**Published:** 2022-12-01

**Authors:** Banafshe Abadi, Nazanin Goshtasbi, Saman Bolourian, Jaleh Tahsili, Mahboubeh Adeli-Sardou, Hamid Forootanfar

**Affiliations:** ^1^ Herbal and Traditional Medicines Research Center, Kerman University of Medical Sciences, Kerman, Iran; ^2^ Brain Cancer Research Core (BCRC), Universal Scientific Education and Research Network (USERN), Kerman, Iran; ^3^ Department of Pharmaceutics, Faculty of Pharmacy and Pharmaceutical Sciences, Tehran Medical Sciences, Islamic Azad University, Tehran, Iran; ^4^ Department of Biology, Faculty of Science, Alzahra University, Tehran, Iran; ^5^ Department of Plant Biology, Faculty of Biological Science, Tarbiat Modares University, Tehran, Iran; ^6^ Medical Mycology and Bacteriology Research Center, Kerman University of Medical Sciences, Kerman, Iran; ^7^ Student Research Committee, Kerman University of Medical Sciences, Kerman, Iran; ^8^ Pharmaceutical Sciences and Cosmetic Products Research Center, Kerman University of Medical Sciences, Kerman, Iran; ^9^ Department of Pharmaceutical Biotechnology, Faculty of Pharmacy, Kerman University of Medical Sciences, Kerman, Iran

**Keywords:** electrospinning, hybrid nanofibers, biomedical applications, wound healing, characterization

## Abstract

Nanotechnology is one of the most promising technologies available today, holding tremendous potential for biomedical and healthcare applications. In this field, there is an increasing interest in the use of polymeric micro/nanofibers for the construction of biomedical structures. Due to its potential applications in various fields like pharmaceutics and biomedicine, the electrospinning process has gained considerable attention for producing nano-sized fibers. Electrospun nanofiber membranes have been used in drug delivery, controlled drug release, regenerative medicine, tissue engineering, biosensing, stent coating, implants, cosmetics, facial masks, and theranostics. Various natural and synthetic polymers have been successfully electrospun into ultrafine fibers. Although biopolymers demonstrate exciting properties such as good biocompatibility, non-toxicity, and biodegradability, they possess poor mechanical properties. Hybrid nanofibers from bio and synthetic nanofibers combine the characteristics of biopolymers with those of synthetic polymers, such as high mechanical strength and stability. In addition, a variety of functional agents, such as nanoparticles and biomolecules, can be incorporated into nanofibers to create multifunctional hybrid nanofibers. Due to the remarkable properties of hybrid nanofibers, the latest research on the unique properties of hybrid nanofibers is highlighted in this study. Moreover, various established hybrid nanofiber fabrication techniques, especially the electrospinning-based methods, as well as emerging strategies for the characterization of hybrid nanofibers, are summarized. Finally, the development and application of electrospun hybrid nanofibers in biomedical applications are discussed.

## 1 Introduction

Currently, nanomaterials do not have an internationally accepted definition. Based on nanomaterials characteristics, they can be designed as nanoparticles (NPs), nanowires, nanotubes, nanofibers, and nanorods (Barhoum et al., 2019a). Recently, nanofibers have drawn significant interest among many researchers in different fields. The unique properties of nanofibers, including high surface area-to-volume ratio, tunable porosity, and superior mechanical and physicochemical properties, make them an ideal candidate for applications that require large surface areas (Yousefzadeh and Ghasemkhah, 2019). Since the production of nanofibers has gained increasing attention in recent years, several conventional scaffold fabrication techniques have been developed. Some of the most commonly used methods include the sonochemical method (Lee et al., 2019b), template-based synthesis (Zhao et al., 2021b), self-assembly (Jiang et al., 2021), electrospinning (Teo and Ramakrishna, 2006; Zahmatkeshan et al., 2019), and polymerization (Bhowmick et al., 2014).

In recent years, electrospinning has gained a great deal of attention to create nanofibers. The major reasons for the popularity of the electrospinning technique are its cost-efficiency, ability to manufacture continuous nanofibers, high flexibility, and simplicity in setting up and controlling nanofiber diameters, compositions, and orientations based on the desired application (Gugulothu et al., 2019). Many materials, such as natural and synthetic polymers, metals, and metal oxides, carbon-based and composite nanomaterials, can be utilized for electrospun nanofiber production (Kenry and Lim, 2017; Barhoum et al., 2019a). Nanofibers have been classified according to their composition (e.g., polymers, metals, metal oxides, ceramics, carbon, and hybrids), size (e.g., diameter, length, porosity), and morphology (e.g., nonporous, mesoporous, hollow, core-shell, biocomponent, multi-component) (Barhoum et al., 2019b).

A wide variety of biodegradable and biocompatible polymers (natural and synthetic polymers) can be combined to create hybrid mats (Khamrai et al., 2019; Ndlovu et al., 2021a). Blending polymers can improve the low mechanical properties of natural polymers and the low biocompatibility of synthetic polymers (Khamrai et al., 2019; Sionkowska, 2021). In addition, different functional agents, such as drugs, biomolecules, and NPs, can be incorporated into the polymeric matrix to produce unique hybrid nanofibers. By combining these materials with polymers in a nanofiber matrix, multifunctional nanocomposites with improved mechanical, chemical, and electrical properties as well as superior biocompatibility and biodegradability, can be created.

In this literature review, the recent research performed on the fabrication and characterization of hybrid electrospun nanofibers, not limited to different blended polymers but considering a broader set of polymers with functional agents, such as drugs, biomolecules, and NPs, is discussed. Also, a summary of the biomedical applications of electrospun hybrid nanofibers in drug delivery, tissue engineering, wound healing, and biosensors is provided. Finally, recent challenges in mechanical strength, degradation, and industrial mass fabrication of electrospun nanofibers, along with prospects of hybrid nanofibers for tissue engineering and biomedical applications, are discussed.

## 2 Hybrid nanofibers fabrication techniques

Hybrid nanofibers have received significant attention owing to their unique properties. These materials show unique magnetic, optoelectrical, and biological properties essential for a wide range of applications in optics, energy generation and storage, environment, medicine, and biotechnology (He et al., 2014; Zeng et al., 2014; Ghajarieh et al., 2021). Various techniques can be used to fabricate hybrid nanofiber-based structures. However, this study has focused on electrospinning methods. These methods enable the production of two-dimensional (2D) as well as three-dimensional (3D) nanofibrous structures, which is of considerable significance. Notably, some exceptional characteristics of hybrid nanofibers have resulted from incorporating functional agents into nanofibrous structures. This section discusses the main electrospinning-based methods to create hybrid nanofibers and immobilization techniques for functional agents, particularly NPs.

### 2.1 Electrospinning methods

Currently, many efforts have been made in the direction of up-scaling the production and improving the nanofiber properties. Among various fabrication techniques, electrospinning is a promising method that offers the opportunity to produce nanofibers using different materials in various fibrous assemblies. In recent years, electrospinning received much attention in both academics and industry due to its simplicity, applicability over a wide range of materials, and low cost (Alghoraibi and Alomari, 2018).

Electrospinning is a dry spinning technique used to fabricate continuous nanofibers (Barhoum et al., 2019b). Fibers are drawn from a melt or liquid polymer solution with electrostatic force, and nanofiber networks are generated in one step (Tucker et al., 2012). It is considered a suitable flexible technique for producing electrostatic fibers on a large scale (Barhoum et al., 2019a; Gugulothu et al., 2019). In this method, the size of fibers can be simply controlled from nanometer to micrometer (Gugulothu et al., 2019).

This technology can generate nanofibers with a high surface-to-volume ratio and a structure similar to the extracellular matrix (ECM), receiving much attention for research in biomedicine (Liu et al., 2021b). Several electrospinning methods have been patented in the past 20 years (Barhoum et al., 2019a). Electrospinning can produce many forms of nanofibers, including smooth nanofibers, branched nanofibers, core-shell nanofibers, ribbon-like nanofibers, porous nanofibers, and nanofibers with fractal surface structures (Yousefzadeh and Ghasemkhah, 2019).

Extensive use of electrospinning in different industries and high-tech fields has led to infrastructure for the mass production of related equipment, making it commercially available alongside nanofibrous material (Blakney et al., 2013). This has made nanofibers available for future clinical use after Food and Drug Administration (FDA) approval (Blakney et al., 2013). There are various electrospinning-based techniques to fabricate 2D and 3D hybrid nanofiber structures for different applications. The conventional electrospinning methods used for 2D nanostructures, including blend electrospinning, coaxial electrospinning, emulsion electrospinning, and side-by-side electrospinning, along with main 3D electrospinning techniques, are described below.

#### 2.1.1 conventional electrospinning methods

##### 2.1.1.1 Blend electrospinning

**FIGURE 1:**
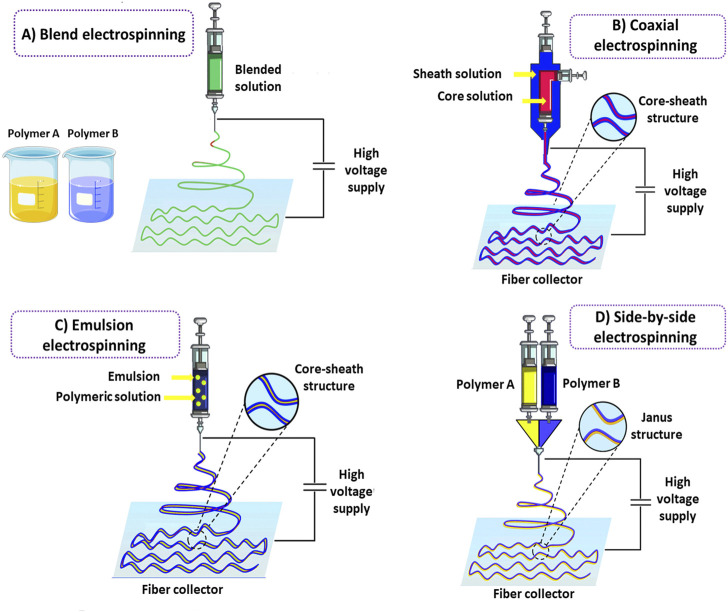
Schematic illustration of conventional electrospinning methods for hybrid nanofibers fabrication. Blend electrospinning **(A)**. Co-axial electrospinning **(B)**. Emulsion electrospinning **(C)**. Side-by-side electrospinning **(D)**.

Blend electrospinning is considered the simplest method, based on mixing different polymers or polymers with functional agents to prepare a single fluid for electrospinning (Figure 1A). In blend electrospinning, drug encapsulation can be achieved in one step by dissolving or dispersing a drug in the polymeric mixture prior to electrospinning. This leads to a prolonged-release profile of the drug under specific conditions. It is noteworthy that the physicochemical properties of polymers strongly affect the functionality and release rate of the encapsulated drug by direct interaction between polymers and drugs. The solubility of a drug is an important issue that should be considered in this method. Insufficient solubility can result in the migration of drug molecules towards the fibers’ surface, leading to the drug’s burst release. This challenge can be overcome by maintaining the balance between hydrophilic and hydrophobic properties between drugs and polymers (Kumar, 2017; Keirouz et al., 2020).

##### 2.1.1.2 Coaxial electrospinning

In order to enhance the efficiency of nanofibers, many modifications have been performed on electrospinning techniques through the years. Developing core-sheath nanofibers utilizing coaxial or triaxial electrospinning is one of these modifications. In this strategy, a polymeric nanofiber in the core is covered by another polymer as a shell (Naeimirad et al., 2018) (Figure 1B). Nanofibers fabricated by the triaxial electrospinning method contain three layers: core, middle, and shell (Yu et al., 2015). Layer by layer, the structure of nanofibers in this method can modify drug profile release and prevent any damage to the drugs incorporated into the core part of the nanofibers. The shell phase can act as a physical barrier, providing prolonged release kinetics and avoiding drug degradation following direct exposure to the external environment (Wang and Windbergs, 2019).

Interestingly, two different release patterns from one delivery system can be reached by incorporating different drugs into the core and shell phases. Coaxial or triaxial electrospinning is the most common technique for preparing hybrid nanofibers. This technique is based on a concurrent flow of a core and sheath solution from separate capillaries to form a nanofiber (Qin, 2017). In this technique, multiple drugs can be incorporated into different nanofiber layers, overcoming incompatibilities (Lu et al., 2016). Furthermore, core-sheath nanofibers made by coaxial electrospinning have higher drug loading efficiency than nanofibers prepared by blend electrospinning, and the possibility of initial burst release is also lower in this kind of nanofibers. The ability of this technique to prepare nanofibers from unspinnable solutions is considered another advantage compared to blend electrospinning (Pant et al., 2019).

##### 2.1.1.3 Emulsion electrospinning

Emulsion electrospinning is one of the most straightforward techniques for preparing hybrid nanofibers. Many scientists have developed nanofibers using emulsion electrospinning to incorporate different therapeutic agents into fibers in the form of core-sheath structures. Emulsion electrospinning has some superiorities compared to traditional techniques, such as allowing the incorporation of lipophilic compounds in low-cost hydrophilic polymers with no need for organic solvents, which are highly limited in food systems. Emulsion electrospinning has a similar setup to blend electrospinning in which immiscible solutions are concurrently spun to produce core-sheath structures. In this method, initially, the emulsification of bioactive agents is performed by adding surfactant into the solution to form a water-in-oil emulsion and thereafter, the as-prepared emulsion is mixed with a polymer solution (Nikmaram et al., 2017). Evaporation of the continuous phase during electrospinning increases the viscosity. The emerging viscosity gradient leads to the migration of the aqueous phase droplets containing bioactive agents towards the jet’s center. The droplets are combined under the electric field resulting from the mutual dielectrophoresis that forms column-like structures and ultimately generates a fiber with a core-sheath structure (McClellan and Landis, 2016; Nikmaram et al., 2017) (Figure 1C). The applied voltage levels, flow rate, and spinning distance significantly affect emulsion-based hybrid nanofibers (Zhang and Wang, 2012). It should be noted that in some cases, the interface tension between the organic and aqueous phases of the emulsion can destroy the bioactive agents incorporated into the electrospun nanofibers (Wang et al., 2012a). Hence, emulsion electrospinning may not be appropriate for loading sensitive bioactive agents into nanofibers.

##### 2.1.1.4 Side-by-side electrospinning

The side-by-side electrospinning technique is a two-compartment system commonly used to prepare Janus nanofibers (Figure 1D). In Janus nanofibers, the composition of the two sides of the structure is different. In contrast to the core-sheath structure, both components directly interact with the surrounding environment, which can be beneficial for developing novel structures. Unlike coaxial electrospinning, two chambers containing polymers are separated in the side-by-side electrospinning strategy. In this method, versatile nanofibers with different widths and interface areas can be developed by designing the structure of the spinneret and regulating the electrospinning parameters (Wang et al., 2020a; Li et al., 2021a). The interaction between fluid dynamics, electrodynamics, and rheology is one of the most challenging issues in the side-by-side synchronization of two fluid flows from the spinneret to the collector under an electrical field (Wang et al., 2019).

#### 2.1.2 3D nanofiber-based scaffolds’ fabrication techniques

Electrospinning not only plays an important role in the construction of 2D nanomaterials, but also in the production of 3D scaffolds. Conventional electrospinning techniques use a charged nozzle containing polymer solution(s) at a certain distance from a static collector and form 2D mats. The 2D electrospinning processes produce tightly packed nanofiber scaffolds with only surface pores due to the sheet-like assembly. These scaffolds have a limited thickness and are unable to infiltrate cells or diffuse nutrients. Thus, researchers try to develop 3D electrospun nanofiber scaffolds to better mimic the ECM’s architecture and morphology. The advantages of electrospinning techniques for fabricating 3D nanofibers include control over morphology and tuning of fiber size and scaffold porosity. Moreover, 3D structures offer a greater surface area than 2D mats, which makes them very promising for various applications (e.g., catalysis, water filtration, energy harvesting, tissue engineering, and drug development) (Radacsi and Nuansing, 2020a). However, the main challenge is scaling up.

Considerable effort has been devoted to fabricating 3D scaffolds, employing auxiliary equipment or modified electrospinning apparatuses. The most commonly used techniques for 3D electrospun scaffolds are multilayer electrospinning, wet electrospinning, template-assisted electrospinning, electrospinning with post-processing (gas-foaming, freeze-drying, and electrospraying), self-assembly electrospinning, and electrospinning combined with 3D printing.

##### 2.1.2.1 *Multilayer electrospinning*

**FIGURE 2:**
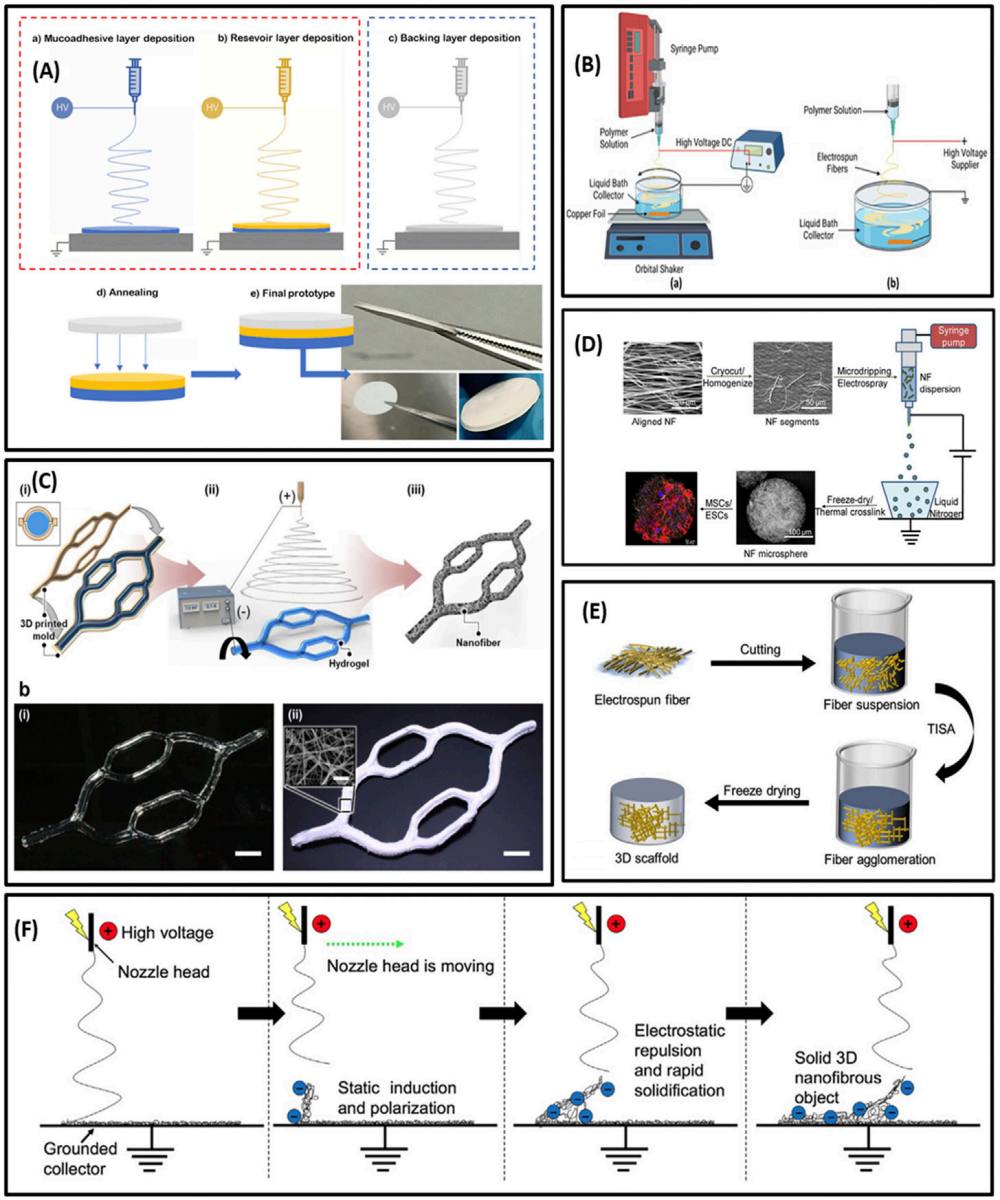
**(A)** Schematic illustration of multilayering electrospinning **(A)**; electrospinning of adhesive layer (a), electrospinning of reservoir layer over the adhesive layer (b); backing layer electrospun separately from the previous layers (c); attachment of the backing layer to the bilayer patch by low-temperature annealing (d); resultant multilayer membrane (e). **(B)** Illustration of wet electrospinning. **(C)** Schematic of template-assisted electrospinning; diagram of template (hydrogel)-assisted electrospinning with the two sequential processes of 3D printing (a–i) and electrospinning (a-ii); photographs of the 3D hydrogel collector (b–i) and the 3D PCL nanofiber macrostructure (bii). **(D)** Schematic summary of nanofiber microspheres fabrication. **(E)** Illustration of freeze-drying post-processing. **(F)** Diagram of self-assembly electrospinning. **(A)** Reprinted with permission from (Teno et al., 2022). Copyright 2022 Multidisciplinary Digital Publishing Institute. **(B)** Reprinted with permission from (Xu et al., 2022). Copyright 2022 Multidisciplinary Digital Publishing Institute. **(C)** Reprinted with permission from (Eom et al., 2020). Copyright 2020 American Chemical Society. **(D)** Reprinted with permission from (Boda et al., 2018). Copyright 2018 American Chemical Society. **(E)** Reprinted with permission from (Yang et al., 2021). Copyright 2021 Multidisciplinary Digital Publishing Institute. **(F)** Reprinted with permission from (Vong et al., 2018). Copyright 2018 Royal Society of Chemistry.

Multilayer electrospinning is one of several promising strategies to fabricate 3D hybrid structures to better mimic the morphological and physicochemical properties of tissues and organs. Multilayer scaffolds can be fabricated by electrospinning nanofibers onto other electrospun fiber mats in a layer-by-layer pattern. Indeed, multilayer electrospinning is the stacking of electrospun layers by sequential electrospinning processes or co-electrospinning (Radacsi and Nuansing, 2020a). Adjusting the fiber diameter, porosity, composition, structure, and mechanical properties of the layers within the scaffold is made possible with multilayer electrospinning. This technology can improve the function of scaffolds for biomedical purposes by enhancing cell adhesion, proliferation, and migration (Sun et al., 2014; Radacsi and Nuansing, 2020a). Therefore, it is highly desirable for tissue engineering, particularly tissues with multilayer structures such as blood vessels and skin (Law et al., 2017). Besides tissue engineering, this approach can be an appropriate choice for developing controlled-release drug delivery systems. For instance, Teno et al. (2022) developed a multilayer system for buccal drug delivery of ciprofloxacin by means of blend electrospinning. In this study, a reservoir layer (ciprofloxacin, poly-ε-caprolactone (PCL) and poly (lactic acid) (PLA)), a mucoadhesive layer (polyethylene oxide (PEO)/PCL/Eudragit RS100) and a backing layer (PCL) were assembled independently to obtain a sustained drug delivery system (Figure 2A). The multilayer strategy exhibited outstanding antimicrobial effects over time and a strong adhesion patch time—for an average of 7 h in volunteers. These results showed the high potential of multilayer electrospun patches as platforms to treat oral infections.

##### 2.1.2.2 Wet electrospinning

Wet or liquid electrospinning is derived from the conventional electrospinning process. In a wet electrospinning process, a liquid/coagulation bath in a metal container is replaced with a solid collector in the electrospinning setup (Figure 2B) (Keirouz et al., 2020). The collector type is essential in electrospinning and significantly impacts the scaffold’s 3D geometry and surface topography. In order to coagulate the electrospun polymers, the liquid bath contains a nonsolvent for the polymers. As the electrospun polymers reach the non-solvent bath, they may precipitate or coagulate. Wet electrospinning can be performed with non-solvent liquids such as water, ethanol, mixed water/ethanol, methanol, tertiary-butyl alcohol, hexane, and subcritical CO2 fluid (Taskin et al., 2020). Hydrophilic polymers such as gelatin, alginate, and chitosan need post-processing to withstand aqueous conditions (Sa and Kornev, 2011). Therefore, post-modifications are required, including surface coating, cross-linking, molding, and freeze-drying with functional moieties in the liquid bath (Jing et al., 2019). Many different 3D hybrid nanofibers like PCL/PEO, cellulose acetate/gelatin have been collected in the coagulation bath by using the wet electrospinning technique (Chen et al., 2019b).

##### 2.1.2.3 *Template-assisted electrospinning*

Template-assisted electrospinning, based on modifying the shape of the collector, is another technique used for fabricating 3D scaffolds. In this method, utilizing an insulated poly (methyl methacrylate) (PMMA) mask on the collecting copper plate to focus the collection of fibers makes it possible to synthesize scaffolds with 3 mm thickness in a short time (Gao et al., 2017). In order to develop customized 3D structures, the architecture of the fiber collector plates may also be modified. Typically, template-assisted collectors are designed by a computer-aided design (CAD) program (Fukunishi et al., 2017) or conventional textiles (Şenel Ayaz et al., 2014). Scaffolds with various shapes such as honeycomb-like (Nedjari et al., 2014), helical spring (Hejazi et al., 2017), metal pin (Kim et al., 2018), and micropatterned structures (Rogers et al., 2014) can be manufactured with this method for different purposes. Additionally, biomimetic collectors, such as auricle-shaped (Walser et al., 2016) and vascular-like (Hammer et al., 2014) collectors, can be designed for tissue engineering applications.

Interestingly, in a recent study, a 3D hydrogel structure as a grounded collector was used instead of the conventional electroconductive collector (Eom et al., 2020). The multi-bifurcated 3D gelatin cylindrical structure was precisely fabricated by 3D printing (Figure 2C). During the electrospinning process, mobile ions in the hydrogel led to concentrating the electric field towards the grounded hydrogel collector and acting like an electroconductive collector. This concentrated electric field permitted the nanofibers to be deposited on the surface of the hydrogel collector, thus electrospun nanofibers precisely replicated the 3D hydrogel collector’s shape and formed a 3D tailored nanofiber macrostructure embedding the hydrogel. Additionally, diverse beneficial features of hydrogels, such as thermally reversible sol−gel transition and exceptional biocompatibility, offer new potential in developing 3D nanofiber macrostructures. For instance, the thermally reversible sol−gel transition of gelatin permitted the elective removal of the 3D hydrogel collector after electrospinning. Furthermore, the remarkable biocompatibility and high-water content of hydrogels eased the loading of biomolecules and cells inside 3D nanofiber macrostructures (Eom et al., 2020).

In some cases, the template may be added to or shaped during the electrospinning process. For example, it has been shown that microcrystals created during low-temperature electrospinning under high humidity can act as removable pore templates between the fibers, leading to the fabrication of highly porous 3D scaffolds. In a similar study, NaCl crystals enhanced nanofibrous scaffolds’ pore size and increased cell proliferation. This technology can also be combined with microfluidic devices to improve cell culture conditions under constant flow (Chen et al., 2016a).

##### 2.1.2.4 Electrospinning with post-processing

3D hybrid scaffolds can also be made from 2D structures using conventional methods followed by post-processing approaches, such as electrospraying, short fiber freeze-drying, gas-foaming, and various other methods. Therefore, post-processing approaches mostly rely on electrospun nanofibers and scarcely need significant changes in the conventional electrospinning setup.

###### 2.1.2.4.1 Gas-foaming

The gas-foaming technique is a simple and versatile post-processing electrospinning approach that enables the conversion of 2D mats to 3D scaffolds. This method is based on *in situ* gas foaming within the pores of the 2D nanofibrous mats as the driving force. In this approach, gas bubbles produced *in situ via* a chemical reaction (e.g., the decomposition of sodium borohydride) or the addition of an inert gas (e.g., carbon dioxide) lead to the creation of highly porous 3D scaffolds (Jiang et al., 2015; Jiang et al., 2018).

Sodium borohydride (NaBH_4_) is the most used foaming agent in the gas-foaming process. However, it needs to be dissolved in water to produce the necessary hydrogen through a chemical reaction with water. Therefore, NaBH_4_ is not applicable to the processing of water-soluble materials. Also, owing to the strong reactivity of NaBH_4_, materials with weak mechanical stability may be destroyed during foaming. In such cases, using carbon dioxide is an appropriate choice for gas-foaming post-treatment. In this process, polymeric material along with carbon dioxide is placed in a chamber at an increasing pressure until the dissolution of the gas in the polymer. When the pressure is relieved, large pores are formed thermodynamically (Pina et al., 2015). This method leads to a highly-interconnected porous structure and significantly enhances the porosity which is beneficial for cell infiltration (Lin et al., 2020; Chen et al., 2021). The porosity and mechanical strength of scaffolds can be modulated by adjusting the chemical reaction’s rate, resulting in gas generation or controlling gas pressure. Also, changing the temperature, pressure, and rates of parameter reduction can control pore sizes (Garg et al., 2012).

###### 2.1.2.4.2 Electrospraying

In addition to aerogels and scaffolds, microspheres can be a promising option for loading various growth factors, cells, and biologics for tissue engineering applications (Hossain et al., 2015). In contrast to implantable scaffolds, injectable microspheres can be utilized to heal irregular defects *via* minimally invasive procedures without the need for invasive surgery (Zhang et al., 2016). Conventional methods for microsphere fabrication (e.g., self-assembly and thermally-induced phase separation) are not applicable to a wide variety of materials (Liu et al., 2011). Hence, alternative approaches for fabricating microspheres are needed.

Nanofibrous microspheres can be prepared by combining electrospraying and electrospinning technology. This strategy is based on the assembly of short electrospun nanofibers into microspheres by electrospraying. For instance, Boda et al. (2018) developed nanofiber microspheres by combining electrospinning with electrospraying. They cut nanofibers into short nanofibers and homogenized them at a low temperature to form a homogeneous dispersion in water. The resulting dispersion was electrosprayed to attain nanofiber microspheres (Figure 2D)**.**

###### 2.1.2.4.3 Short nanofiber assembly (freeze-drying)

Using short electrospun nanofibers as building blocks for constructing 3D scaffolds is a promising approach to adjusting the physicochemical properties and mechanical flexibility of the scaffolds. In this method, electrospun fibers are initially cut into short fibers and homogeneously dispersed in a solution using an ultrasonic homogenizer. Afterward, the solution containing short nanofibers is freeze-dried to prepare highly porous 3D sponges or aerogels (Chen et al., 2016b) (Figure 2E). Different cross-linking strategies can be applied to regulate the mechanical properties and biological functions of the scaffold. Cross-linking among short fibers can be accomplished by chemical cross-linking or temperature-mediated cross-linking. In chemical cross-linking, the functional groups on the surface of nanofibers are exploited to form cross-linking agents-mediated covalent bond formation, whereas in temperature-mediated cross-linking, increasing the temperature can lead to thermal annealing mediated physical cross-linking among short nanofibers (Chen et al., 2022).

##### 2.1.2.5 Self-assembly electrospinning

Since desired structures can be created in a single step, 3D nanofibrous scaffolds self-assembly *via* electrospinning is of interest. In this process, 3D structures result from the fast solidification of the nanofibers, leading to a self-standing object (Figure 2F). This incidence also depends on electrostatic induction and polarization of the deposited nanofibers. Therefore, charging the top part of the deposited nanofiber mats to negative *via* an electric field is necessary for providing a favored deposition area to attract the positively charged jet ejected from the electrospinning nozzle. Considering the negative charge of the fibers in the top part of mats, they will repel each other, leading to the creation of 3D spongy scaffolds (Sun et al., 2012; Mi et al., 2017).

The structure and pore size of scaffolds can be controlled by modulating the polarizability of electrospun fibers by including some conductive additives such as phosphoric acid (H_3_PO_4_) in the polymeric solution. In other words, adding such compounds to the polymeric mixtures can induce repulsive forces between nanofibers during the electrospinning process, resulting in 3D porous structures (Chin and Chang, 1989; Farrokhi-Rad, 2016). It is noteworthy that manipulating the electric field can also affect the structures of resulting 3D mats. For instance, in a recent study performed by Yan et al. (2011), the self-assembly of PCL electrospun nanofibers into 3D honeycomb structures was explored. In order to interpret this incidence, they hypothesized that it may be related to the gradients in the applied electric field.

##### 2.1.2.6 Combining 3D printing with electrospinning

3D bioprinting or additive manufacturing (AM) technology can bridge the gap between artificial tissue scaffolds and natural tissues (Zhang et al., 2017b). 3D printing improves scaffolds’ pore size, interconnections, and mechanical strength by layering the materials together (Yang et al., 2022). This technique has been used in various technical and biomedical applications, including medical and aviation devices (composite, metal, or plastic), personalized clothes, and grafts (Su et al., 2021). The 3D printing technique comprises three basic components: hardware (the 3D printer), software, and materials.

Additionally, CAD-based 3D printing technology has recently gained considerable attention, allowing the fabrication of cellular, acellular, and hybrid scaffolds (Abel et al., 2020). 3D bioprinting has made major advances in recent years. However, it is still associated with many limitations, hindering its clinical application. The most crucial challenge is the low mechanical strength of 3D printed scaffolds, which restrict their biomedical application, especially in hard tissue engineering. Furthermore, the variety of materials, and the resolution of scaffolds that can be printed, are limited (Potyondy et al., 2021; Yang et al., 2022).

To overcome this challenge, the combination of 3D printing with electrospinning can produce multifunctional hybrid nanofibers with highly porous interconnected structures and improved mechanical properties (Yang et al., 2022). Different methods for combining these two techniques include electrospinning onto 3D printed scaffolds, 3D printing onto electrospun fibers, alternate use of 3D printing and electrospinning, 3D printing with short fiber inkgenerated from electrospun nanofibers, decorating/infusing 3D printed scaffolds with electrospun nanofiber segments, fabrication of electrospun scaffolds on 3D printed collectors/templates, combination al use of different components prepared by electrospinning and 3D printing, electrohydrodynamic (EHD) printing, and a platform combining 3D printing and electrospinning techniques (Yang et al., 2022).

### 2.2 Functionalization of nanofibers

The properties of hybrid nanofibers made by blending only polymers are limited, and much can be performed to improve their biological, mechanical, electrical, or optical features. The mentioned properties can be improved by immobilizing functional agents in nanofibers and forming multifunctional hybrid nanofibers. These functional agents include inorganic/organic NPs, biomolecules (e.g., growth factors, hormones, and nucleic acid), drug molecules, etc. The immobilization of various functional moieties in nanofiber matrix can considerably improve mechanical, physicochemical, and biological properties (Foraida et al., 2017; Nirwan et al., 2022).

#### 2.2.1 Functionalization using nanoparticles

Immobilization of NPs in hybrid nanofibers provides exceptional properties, combining NPs’ advantages with polymer properties. These nanofibers may also have more functions, including photothermal properties, magnetic responses, biosensing, antibacterial properties, and drug delivery capabilities. Furthermore, NPs can improve nanofibers’ physicochemical and mechanical properties and stability (Kalia and Haldorai, 2015).

An increasing number of published articles on nanofiber-nanoparticle hybrids show the high potential of such multifunctional nanofibers for different applications (Kalia and Haldorai, 2015). Diverse types of NPs have successfully been used for this aim, including metal (Li et al., 2017), metal oxide (Pinto et al., 2016), carbon nanotubes, and polymeric NPs (Tuğcu-Demiröz et al., 2021).

The functionalization of nanofibers with NPs can be achieved using different methods, including direct solubilization of the NPs in an electrospinning solution (Wang et al., 2018a), reduction of the precursor for NPs *in situ*, growth of NPs on nanofibers (Zhang et al., 2018), or electrospraying NPs on nanofibers’ surfaces (Nekounam et al., 2020).

Co-blending NPs with a polymer solution is the most common method to fabricate nanofiber-nanoparticle hybrids. In this method, NPs will be uniformly distributed on the surface or inside the nanofibers. Notably, during this process, the interfacial interaction between NPs and polymer solution can strongly influence the spinnability, composite fiber morphology, and mechanical properties. The aggregation of NPs in the working solution can lead to needle blocking during the electrospinning process, and NPs will not be distributed in the nanofibers, reducing the mechanical properties and functionality of composite fiber membranes. To minimize this effect, NPs can be combined with a polymer solution through stirring and sonication, separately dissolving the NPs and polymer in different solvents or adding a certain amount of surfactant into the working solution (Rasekh and Raisi, 2021; Jiang et al., 2022; Ren et al., 2022).

In addition to the co-blending strategy, NPs can be loaded into nanofibers by combining other processes with electrospinning technology. For instance, combining electrospinning with plasma technology can lead to the formation of NPs on the surface of nanofibers containing the precursor for NPs (Annur et al., 2015). The electrospray or magnetron sputtering technology also allows the uniform coverage of NPs on the surface of nanofibers (Enculescu et al., 2021; Park et al., 2021).

Multifluid electrospinning, such as coaxial or side-by-side electrospinning, can also be used for preparing nanofiber-nanoparticle hybrid scaffolds. In these methods, polymer solution and NPs are loaded into separate syringes to reduce the possibility of NPs aggregation inside the polymer solution (Zhang et al., 2022a).

## 3 Effective parameters in electrospinning

Different parameters can influence the electrospinning process. Therefore, adjusting these parameters is essential to achieve desired nanofibers. The main effective parameters in the electrospinning process include: 1) physicochemical properties of the system (nature of the polymer, solution viscosity, conductivity, and surface tension) (Madruga and Kipper, 2022), 2) process parameters (voltage, receiving distance, and flow rate) (Zhang et al., 2022b), and 3) environmental factors (temperature and humidity) (Wang et al., 2021b). The mentioned parameters’ effects are presented in Table 1.

**TABLE 1:** Effect of different parameters on the electrospinning process (Zhang et al., 2022a).

	Influence factor	Influence results	Ref
System parameters	Polymer concentration	Increased polymer concentration or molecular weight leads to greater entanglement between molecule chains and enhanced intermolecular Coulomb forces, which can increase the fiber diameter	Filip et al. (2021)
	The molecular weight of polymers		(Bai et al., 2021; Li et al., 2021c)
	Surface tension	Enhancing the droplet’s surface tension leads to the jet expending more energy to offset this negative effect. The speed of the jet slows down, requiring more time to stretch the fibers, leading to a decrease in the fiber diameter	Fan et al. (2021)
	Conductivity	Increased conductivity leads to charge accumulation on the surface of the jet. The fibers stretch more quickly in this state and the diameter of the fiber is decreased. However, the Coulombic repulsion at the jet interface is augmented when the solution conductivity is increased further Indeed, the unstable bending whip effect causes uncontrollable fiber diameter distribution	(Ma et al., 2021; Silva et al., 2021; Topuz et al., 2021)
Process parameters	Voltage	Increased voltage enhances the charge density on the jet’s surface, leading to a significant effect of jet stretching. Consequently, the fiber diameter declines and the fibers’ crystallinity is improved. However, the flow rate at the spinneret is amplified in too high voltage. This can increase the diameter of the fiber	Joy et al. (2021)
	Flow rate	The rise of the flow rate increases the solution at the spinneret, increasing the fiber’s diameter and pore size. When the flow rate is too fast, the solution’s gravity causes it to trickle straight down and form beaded fibers	Lei et al. (2020)
	Receiving distance	The additional receiving distance gives the jet more time to extend, leading to the shrinkage of the fiber’s diameter	Wang et al. (2021a)
Environmental factors	Temperature	Increased temperature reduces the viscosity of the solution and intermolecular Coulomb force, decreasing the fiber diameter	Kopp et al. (2020)
	Humidity	When humidity is too high, fiber production is faster. Water droplet condensation on the fiber surface leads to wrinkles occurring on the surface of the fibers. At low humidity, the removal of the solvent from the tip of the needle is slower than the evaporation rate of solvent, leading to the clogging of the needle tip and stopping electrospinning	Wu et al. (2021)

## 4 Polymers in electrospun nanofibers

Various polymers from different sources can be combined and spun to fabricate hybrid nanofibers. Selection of the polymers is a critical step for producing nanofibers with specific characteristics suited to particular applications. The ideal polymer for biomedical applications must be biodegradable, safe, biocompatible, moderately hydrophilic, and have proper mechanical strength. These polymers can be obtained from natural or synthetic sources, each having different advantages and disadvantages. The polymer type must be selected based on the end-use of the nanofibers (Makadia and Siegel, 2011). Table 2 lists the polymers most commonly used in nanofiber production.

**TABLE 2:** Summary of natural and synthetic polymers for nanofiber fabrication.

	Polymer	Biodegradability	Advantage	Disadvantage	Ref
Natural polymers	Chitosan	Fast-biodegradable	✓Biocompatible		(Kalantari et al., 2019; Iacob et al., 2020; Juncos Bombin et al., 2020; Khosravimelal et al., 2021)
			✓Non-toxic		
			✓Antibacterial		
			✓Support hemostasis	✗Poor electrospinnability	
			✓Accelerating wound healing process	✗Poor mechanical strength	
	Cellulose	Fast-biodegradable	✓Biocompatible		Riva et al. (2021)
			✓Non-toxic		
			✓Proper mechanical strength		
			✓Relative thermally stable		
			✓High sorption capacity		
	Silk	Slow-biodegradable	✓Biocompatible		(Huang et al., 2018; Khosravimelal et al., 2021)
			✓Low toxicity	✗Batch-to-batch variation	
			✓Proper mechanical property	✗Difficult scale-up processing	
			✓Toughness and elasticity		
	Gelatin	Slow-biodegradable	✓Biocompatible		Ndlovu et al. (2021b)
			✓Non-toxic		
			✓Low antigenicity	✗Poor mechanical strength	
			✓Promoting cellular attachment and growth		
	Collagen	Slow-biodegradable	✓Biocompatible		Hernández-Rangel and Martin-Martinez, (2021)
			✓Non-toxic	✗Poor thermal stability	
			✓Promoting cellular attachment and growth	✗Poor stability in solvent	
				✗Poor mechanical strength	
	Alginate	Fast-biodegradable	✓Biocompatible		(Rastogi and Kandasubramanian, 2019; Wilk and Benko, 2021)
			✓Low toxicity	✗Poor electrospinnability	
			✓Antimicrobial		
			✓High hygroscopicity		
			✓High ion adsorption		
	Hyaluronic acid	Fast-biodegradable	✓Biocompatible		(Bayer, 2020; Iacob et al., 2020; Castro et al., 2021)
			✓High swelling and water absorption	✗Poor electrospinnability	
			✓Non-immunogen		
	Starch	Fast-biodegradable	✓Biocompatible		(Rodrigues and Emeje, 2012; Liu et al., 2017; Amaraweera et al., 2021)
			✓Non-toxic	✗Poor thermo-mechanical properties	
			✓Inexpensive		
Synthetic polymers	PVA	Fast-biodegradable	✓Biocompatible		Huang et al. (2019)
			✓Non-toxic		
			✓Biologically stable		
			✓Flexible mechanical property		
	PLA	Slow-biodegradable	✓Biocompatible		Saini et al. (2016)
			✓Processable	✗Hydrophobic nature	
			✓Favorable mechanical property	✗Low toughness	
	PCL	Slow-biodegradable	✓Biocompatible	✗Poor wetting surface	(Rashtchian et al., 2020; Murugan and Parcha, 2021)
			✓Excellent mechanical strength	✗Preventing cell adhesion and proliferation	
	PU	non-biodegradable	✓Biocompatible		(Shin and Choi, 2018; Movahedi et al., 2020)
			✓Excellent mechanical strength	✗Hydrophobic surface	
			✓Good barrier properties and oxygen permeability		
			✓Elastomer-like character		

PVA: Poly (vinyl alcohol), PLA: Poly(lactic acid), PCL: Poly(ε-caprolactone), PU: Poly(urethane).

Natural polymers are a popular choice in electrospinning for biomedical applications. They can come from animal or plant resources or other living organisms, making them mostly biodegradable, biocompatible, and non-toxic with low antigenicity (Juncos Bombin et al., 2020). Additionally, they may have biological effects such as antimicrobial activities, anti-inflammatory, and hemostatic effects. Compared with synthetic polymers, natural polymers are more costly and harder to process during electrospinning (Juncos Bombin et al., 2020). It is noteworthy that synthetic polymers have better thermal stability, electrospinning, and mechanical properties. Synthetic polymers are favored over bio-based polymers for specific applications, as they can be applied to develop nanofibers with optimum mechanical and degradation characteristics. They can be combined with natural polymers to adjust their mechanical performance and degradation rate (El-Aassar et al., 2021).

## 5 Characterization of a hybrid nanofiber

The high quality of nanofibers can be assured during the production process by characterizing nanofibers according to test metrics that correlate with the material’s functional properties. Single fiber characterization provides fundamental data for understanding the relationship between nanofiber’s structure and properties. Also, depending on their ultimate utilization, various methods have been developed to characterize nanofiber scaffolds (Lopez Marquez et al., 2022). For example, electrical conductivity and electrochemical reactivity are necessary when nanofibers are intended to be used as sensors (Liu et al., 2019). If the nanofibers are synthesized for air filtration, fibers’ permeability, porosity, and particle penetration are significant characteristics (Lu et al., 2021). In the following section, we summarize various characterization techniques in order to gain a better understanding of nanofibers’ function.

### 5.1 Morphological characterization

#### 5.1.1 Porosity

Porosity refers to the empty spaces between nanofiber components. These pores are located on the surface or inside of nanofibers, making them very lightweight and providing a large surface area (Roodbar Shojaei et al., 2019; Yousefzadeh and Ghasemkhah, 2019). Porous nanofibers can be used in the fields of filtration, gas separation, energy, sensor, and tissue engineering. According to the American Society for Testing and Materials (ASTM), the porosity of nanofibers can be measured by different methods such as mercury porosimetry, liquid extrusion porosimetry, capillary flow porometry (CFP), and nuclear magnetic resonance (NMR) (Roodbar Shojaei et al., 2019). Porosity is an important parameter when selecting the scaffold for tissue engineering. In the study conducted by Ghasemi-Mobarakeh et al. (2008), the porosity of hybrid nanofibers (PCL/gelatin) was calculated by the CFP method. According to the results, the pore diameter decreased with increasing gelatin content. In another study, the porosity of 3D scaffolds manufactured by co-electrospinning of poly (hydroxybutyrate-co-hydroxy valerate (PHBV)/PCL), as the first layer, and diatom shells (DS) incorporated pullulan (PUL), as the second layer, was measured by mercury porosimetry. The calculated value was 42.17% (Dalgic et al., 2019).

#### 5.1.2 Diameter and size distribution of electrospun nanofibers

Geometric characterizations are able to determine the diameter, size distribution, orientation, and morphology of the fabricated fibers. Morphological features of nanofibers can be measured by scanning electron microscopy (SEM), energy dispersive X-ray spectroscopy (EDX), atomic force microscopy (AFM), and transmission electron microscopy (TEM).

SEM is the most commonly used method due to its availability and ease of use. By measuring apparent density from the SEM images and comparing it to the bulk density of the electrospun polymers, SEM can reveal information about the fiber diameter/alignment, pore diameter, and porosity of scaffolds (Lopez Marquez et al., 2022). In conjunction with SEM, an EDX is also used to analyze chemical composition near the sample’s surface (Tomlins, 2015). However, there is a probability of accuracy reduction for fibers with <200 nm in diameter because the electron beam can destroy these fibers. Moreover, coating non-conductive samples with conductive metals may result in questionable accuracy in thin fibers. Based on the mentioned reasons, atomic force microscopy (AFM) or transmission electron microscopy (TEM) are better options for determining the morphology of nanofibers, especially tiny fibers (Ramakrishna, 2005). AFM provides information about the surface of the nanofiber’s topographical, morphological, mechanical, and physicochemical properties (Lopez Marquez et al., 2022). It has been reported that scaffold stiffness can modulate cell function and remodeling (Zhu et al., 2019; Yi et al., 2022). AFM can be used to measure and interpret scaffold stiffness; thus, it can be helpful in tissue engineering and regenerative medicine. TEM imaging is a leading method for studying hybrid nanofibers, especially core-sheath nanostructures. This method is based on different amounts of electron transmission by each polymer. It can be used on individual fibers, especially for fibers containing nanomaterials (Lopez Marquez et al., 2022).

### 5.2 Mechanical characterization of the nanofibers

#### 5.2.1 Tensile

The tensile test is one of the most frequently used methods for determining nanofiber’s mechanical properties. Tensile tests define factors such as young’s modulus, tensile strength, and strain at the break of polymeric fibers. Previous studies have reported that hybrid scaffolds exhibit better mechanical strength (Karbasi et al., 2016; Hou et al., 2019).

### 5.3 Structural evaluation of nanofibers

#### 5.3.1 X-ray diffraction (XRD)

XRD spectroscopy is a non-destructive and well-established method to detect changes in crystallinity after electrospinning. The crystallinity of many different hybrid electrospun scaffolds has been clarified using the XRD method (Yuan and Zhang, 2012; Manjumeena et al., 2015; Yang et al., 2015; Lv et al., 2021). The crystallization level can explain the relationship between fiber’s diameter and drug encapsulation efficiency in core-sheath electrospun nanofibers (He et al., 2015). Since the drug/polymer system has a limited time to recrystallize during electrospinning, non-crystalline solid dispersions are more likely to form (Preem et al., 2017). Amorphous solid dispersions of drugs are commonly confirmed using the XRD method after blend electrospinning (Lopez et al., 2014; Tamm et al., 2016; Preem et al., 2017).

### 5.4 Chemical characterization of nanofibers

#### 5.4.1 Fourier transform infra-red (FTIR)

Information about nanofiber composition and chemical characterizations can be gleaned from FTIR analysis (Huang et al., 2000). Furthermore, FTIR can be used to detect inter and intramolecular bonding, polymer-polymer, and polymers-drug interactions (Preem et al., 2017; Zhao et al., 2021a). Additionally, the effect of surface functionalization (Karuppannan et al., 2022) and cross-linking (Baştürk and Kahraman, 2012; Jia et al., 2022) of hybrid nanofibers can be evaluated by this method.

#### 5.4.2 Raman spectroscopy

Raman spectroscopy is typically complemented by infrared (IR) spectroscopy (Horzum et al., 2019). Raman spectroscopy has been widely used to investigate the considerable variations and other low-frequency modes in the distribution of polymers within polymeric nanofiber matrices and to assess the effects of carbonaceous or nanostructured materials on the morphology and physical properties of the electrospun nanofibers (Chipara et al., 2013; Kotzianová et al., 2016; Roodbar Shojaei et al., 2019).

#### 5.4.3 Water contact angle measurement

Wettability or hydrophilicity of the surface is important for understanding the outer surface chemistry of electrospun nanofibers. Of note, the hydrophilicity of nanofiber surface is vital in biological studies due to better cell attachment and proliferation (Unnithan et al., 2014; Kalia and Haldorai, 2015).

### 5.5 Thermal evaluation techniques

Thermal properties of hybrid electrospun nanofibers can be evaluated by differential scanning calorimeter (DSC) and thermogravimetric analysis (TGA) methods (Aykut et al., 2019).

#### 5.5.1 Differential scanning calorimeter (DSC)

DSC consists of a cooling or heating test to maintain the sample and reference at a constant temperature. The DSC can be used to determine the solid-state properties of active pharmaceutical agents and the encapsulation efficiency of crystalline compounds and identify the configuration of the macromolecules in nanofibers and their interactions (e.g., plasticizing and hydrogen bonding) (Balogh et al., 2014; Lopez et al., 2014; Deng et al., 2018; Islam et al., 2019).

#### 5.5.2 Thermogravimetric analysis (TGA)

TGA is employed to determine the encapsulation efficiency of hybrid nanofibers, the stability, and the solvents evaporation of formulations by measuring material weight (loss or gain) as a function of altering the heating temperature (Waghmare et al., 2018; Islam et al., 2019; Ye et al., 2021).

## 6 Biomedical applications of hybrid nanofibers

**FIGURE 3:**
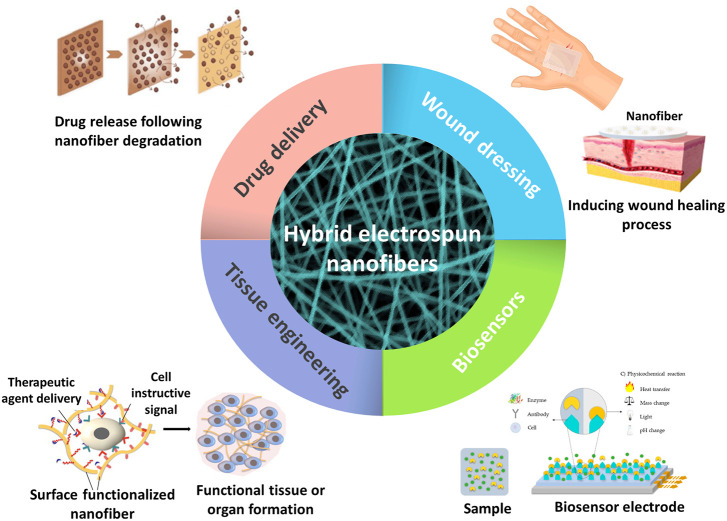
Various applications of hybrid electrospun nanofibers in biomedical field.

Biocompatibility and mechanical properties are important parameters in biomedical applications. The previously mentioned positive characteristics of electrospun nanofibers, high porosity, versatility, and high surface-to-volume ratio, make them a good choice for different biomedical applications such as drug delivery, tissue engineering, wound dressing, and biosensors (Liu et al., 2021b) (Figure 3).

### 6.1 Drug delivery

To date, various drug delivery systems have been developed to enhance the clinical efficiency of drugs and decrease their toxic effects compared to conventional dosage forms. Some characteristics of an ideal drug delivery system include high loading capacity and encapsulation efficiency, ease of operation, low cost, and controlled drug release. All of the mentioned criteria can be achieved using electrospun nanofibers (Torres-Martinez et al., 2018).

Electrospun nanofibers have received a great deal of attention among different drug delivery systems due to their favorable characteristics. A wide variety of materials and drugs, ranging from small molecule drugs, such as antibiotics, to macromolecules, like proteins and DNA, can be loaded into electrospun nanofibers for improving their bioavailability or attaining controlled release (Torres-Martinez et al., 2018). Different factors can affect the drug release from nanofibers which should be considered in designing drug-loaded nanofibers. For instance, the degradation and dissolution rate of the polymeric matrix can significantly influence drug release, which is associated with water solubility of components and structural properties of nanofibers such as fiber diameter, specific surface area, size and total volume of pores, and crystallinity of polymeric matrix. The nature of the drug and its compatibility with the matrix should also be taken into consideration as one of the practical factors in the drug release profile (Hrib et al., 2015).

In recent years, a large part of studies in the area of controlled-release drug delivery has focused on developing stimuli-responsive systems. The drug release in these systems is based on response to changes in environmental parameters, including pH, temperature, light, electrical, and magnetic fields (Contreras-Cáceres et al., 2019). Electrospun nanofibers are appropriate candidates for developing stimuli-responsive systems because they can be easily manipulated to tailor the drug release rate. In the following section, an overview of drug-loaded hybrid nanofibers is presented. Table 3 provides a summary of electrospun hybrid nanofibers used for drug delivery.

**TABLE 3:** An overview of electrospun hybrid nanofiber for drug delivery.

Drug	Class	Polymer	Study	Electrospinning method	Application	Ref
**Ibuprofen**	Anti-inflammatory	Poly (N-isopropyl acrylamide)/PCL	*-*	Blending	✓Inflammatory disorders	Tran et al. (2015)
**Ibuprofen**	Anti-inflammatory	PCL/PVA/COll	*In vitro*	Blending	✓Guided tissue engineering	Limoee et al. (2019)
**Dexamethasone**	Anti-inflammatory	PLA/PCL	*In vitro*	Blending	✓Tissue engineering	Vacanti et al. (2012)
**Ketoprofen**	Anti-inflammatory	Poly (N-isopropyl acrylamide)/EC	*In vitro*	Blending	✓Tissue engineering	Hu et al. (2016)
**Rhodamine B**	Anti-inflammatory	PVA/POX	*In vitro*	Blending	✓Inflammatory diseases	Phromviyo et al. (2015)
**7-Ethyl-10-amino-hydroxy camptothecin**	Anti-inflammatory	PCL/gelatin	*In vitro*	Blending	✓Glioma	Zhu et al. (2015)
**Cisplatin**	Anticancer	PCL/CS	*In vitro*	Blending	✓Cervical cancer	Aggarwal et al. (2017)
			*Ex vivo*			
			*In vivo*			
**Doxorubicin hydrochloride**	Anticancer	PLA/PEO	*In vitro*	Blending	✓Hepatic cancer	Kuang et al. (2018)
			*In vivo*			
**PTX and DOX hydrochloride**	Anticancer	PEG/PLA	*In vitro*	emulsion-electrospinning	✓Glioma	Xu et al. (2009)
**Camptothecin and DOX loaded mesoporous ZnO (DOX@mZnO)**	Anticancer	PLGA/gelatin	*In vitro*	Blending	✓Liver cancer	Wei et al. (2014)
**Cisplatin and Curcumin**	Anticancer	PLA/PEO	*In vitro*	Blending	✓Cervical cancer	Ma et al. (2015)
			*In vivo*			
**5-FU**	Anticancer	PLA/Keratin	*In vitro*	Blending	✓Colorectal cancer	Zhang et al. (2020)
**Tetracycline**	Antibiotic	Thermoplastic CMC/PEO	*In vitro*	Core-Shell	✓Potential as antibacterial materials for tissue engineering and pharmaceutical science	Esmaeili and Haseli, (2017)
**Ciprofloxacin**	Antibiotic	PVA/Alginate	*In vitro*	Blending	✓Wound dressing	Kataria et al. (2014)
			*In vivo*			
**Ofloxacin**	Antibiotic	Gellan/PVA	*In vitro*	Blending	✓Gastroretentive sustained drug release system for ofloxacin	Vashisth et al. (2017)
			*In vivo*			
**Ofloxacin**	Antibiotic	CS/PVA and Eudragit RL100	*In vivo*	Blending (Multi-layer)	✓Potential as ocular drug delivery systems	Mirzaeei et al. (2021)
**Linezolide**	Antibiotic	PLGA/PCL	*In vitro*	Blending	✓Prophylaxis of skeletal prosthesis-related infections	Eren Boncu et al. (2020)
			*In vivo*			
**Moxifloxacin**	Antibiotic	PVA/CS	*In vitro*	Blending	✓Potential as antibacterial materials	Liu et al. (2021a)
**Doxycycline in core and shell**	Antibiotic	PVA/PCL	*In vitro*	Coaxial electrospinning	✓Potential in enhancing implant osseointegration and preventing infection	Song et al. (2017)
			*In vivo*			
**Doxycycline**	Antibiotic	CS/PVA	*In vitro*	Blending	✓Diabetic wounds	Hedayatyanfard et al. (2020)
			*In vivo*			
**Vancomycin**	Antibiotic	PVA/graphene oxide sheets/PCL	*In vitro*	Coaxial electrospinning	✓Antibacterial material with time-programmed, biphasic release behavior	Yu et al. (2016)
**Nicorandil**	Vascular vasodilator	HA/PVA	*In vitro*	Blending	✓Potential for the sublingual administration of antianginal drugs	Singh et al. (2016)
			*In vivo*			
**Simvastatin**	antilipemic agent	CS/β-cyclodextrin	*In vitro*	Blending	✓Restenosis prevention	Kersani et al. (2020b)
**Carvedilol**	Β-blocker	PVP/PEG	*In vitro*	Blending	✓Carvedilol sublingual delivery	Wang et al. (2020b)
**Heparin**	Anticoagulant	PLCL/heparin/silk	*In vitro*	Bi-layer	✓Small-caliber blood vessel grafts to treat cardiovascular diseases	Kuang et al. (2020)
			*In vivo*			
**Azithromycin**	Antibiotic	CS/PVA/PVP	*In vitro*	Blending	✓Prolonged ophthalmic delivery of azithromycin	Taghe et al. (2022)
			*In vivo*			
**Ferulic acid, an antioxidant, ε-polylysine**	Antioxidant and antibacterial agent	Hyaluronan/PVP	*In vitro*	Blending	✓Potential for ocular drug delivery	Grimaudo and Concheiro, (2020)

PCL: polycaprolactone, PVA: polyvinyl alcohol, Coll: Collagen, PLA: polylactic acid, EC: ethyl cellulose, POX: polyoxalate, CS: chitosan, PEO: polyethylene oxide, PEG: polyethylene glycol, CMC: carboxymethyl cellulose, PLGA: Poly(lactide-co-glycolide), HA: hyaluronic acid, PLCL: Poly(L-lactide-co-ε-caprolactone), PVP: polyvinylpyrrolidone, DOX: doxorubicin, 5-FU: 5-fluorouracil.

#### 6.1.1 Anti-inflammatory drug

Anti-inflammatory agents refer to compounds that can suppress inflammation and swelling symptoms and may have analgesic and antipyretic effects. Loading anti-inflammatory agents into nanofibers has been of great interest for researchers due to the low water-solubility of such molecules and the desired effect of almost immediate relief. In a recent study, nanofiber-based scaffolds were prepared with a polymeric mixture (PLA and PCL) and dexamethasone, the steroid anti-inflammatory drug, to decrease immune response in tissue engineering. They showed a controlled release of dexamethasone over time, and stem cells could also successfully attach and proliferate on the nanofibers *in vitro* (Vacanti et al., 2012). In another study, electrospun nanofibers were developed as Guided Tissue Regeneration (GTR) membrane by PCL and a blend of polyvinyl alcohol (PVA)/collagen/Ibuprofen (Ibu) (Limoee et al., 2019). The membrane indicated satisfying mechanical properties, and Ibu release was sustained and controlled. In a recent study, to achieve pH and thermo-responsive release of ketoprofen, poly (N-isopropyl acrylamide) (PNIPAAm), as a thermo-sensitive polymer, and Eudragit^®^ L100-55 (EL100-55), as a pH-sensitive polymer, were used to prepare electrospun nanofibers (Li et al., 2018). They showed that the release of ketoprofen was dependent on pH and temperature.

#### 6.1.2 Antibiotics

Microbial infection is considered one of the most challenging issues in medicine that currently threatens world health. In addition, antimicrobial resistance is accelerating due to the overuse of antibiotics. Therefore, designing an efficient drug delivery system for antibiotics with selective action in the infection site to prevent overdosage and antimicrobial resistance is necessary. Eren Boncu et al. (2020) prepared electrospun nanofibers composed of poly (lactic-co-glycolic acid) (PLGA) and PCL loaded with linezolid for prophylaxis of skeletal prosthesis-related infections. They showed that nanofibers could effectively improve healing in damaged and infected areas in the rat model by providing an optimal dosage of the drug in the intended site. They could reduce the need for antibiotic administration by around 37-fold compared to usual methods by providing controlled drug release. This approach is a cost-effective treatment that can avoid the progression of antibiotic resistance (Eren Boncu et al., 2020). In a recent study, nanofibers were fabricated by electrospinning a blend of PVA and chitosan incorporated with moxifloxacin (Liu et al., 2021a). According to the obtained results, the as-prepared nanofibers could significantly inhibit the growth of *Staphylococcus aureus* and *Pseudomonas aeruginosa.* They possessed better antibacterial activity than the control group (Liu et al., 2021a).

#### 6.1.3 Antitumor drugs

Despite all the advances in oncology, cancer is among the deadliest diseases in the world. Cancer treatment by chemotherapy is generally based on the administration of cytotoxic agents to suppress the growth of cancerous cells. It is clear that severe adverse effects accompany the administration of cytotoxic drugs. Thus, developing a localized drug delivery system can be a promising approach to promote the efficiency of conventional chemotherapeutic agents by restricting the action of drugs mostly to tumor tissues and reducing systemic adverse effects. Tumors have specific features that can be considered for developing delivery systems with high specificity towards cancer cells. For instance, due to the increased metabolic rate in tumor cells and high lactic acid production resulting from the glycolytic pathway, the tumor microenvironment is acidic compared to normal tissues. Hence, the pH value difference can be taken into consideration for the fabrication of responsive materials to release loaded cargos only in an acidic environment such as tumors. Zhang et al. (2020) designed a pH-responsive scaffold based on electrospun nanofibers for 5-fluorouracil (5-FU). To achieve pH-responsive release of the drug, 5-FU was covalently attached to keratin, and the resulting polymer was blended with PLA for electrospinning and preparing nanofibrous scaffolds for localized delivery of 5-FU. Fabricated scaffolds showed potent antitumor effects following a rapid release of drugs in the first hours. However, it could not provide continuous release of the drug for a prolonged time. Notably, the prolonged release of the cargo in the tumor site is necessary to eradicate the tumor (Zhang et al., 2020).

Carbon nanotubes (CNTs) have the potential to be utilized for improving mechanical, structural, and drug delivery properties in electrospun nanofibers. In a recent study performed by Qi et al. (2016), doxorubicin (DOX) was chemically attached to the surface of multi-walled carbon nanotubes (MWCNTs). After optimization, the drug was encapsulated up to 83.7%, and as-prepared MWCNTS@DOX particles were blended with a PLGA polymer solution to synthesize a hybrid nanofiber by electrospinning. The incorporation of MWCNTs into PLGA nanofibers not only did not change the morphology of the PLGA nanofibers, but also enhanced their mechanical properties. In addition, this hybrid system could decrease burst DOX release and provide sustained release of DOX over 42 days. One of the strategies to kill cancer cells is increasing temperature locally in the tumor tissue, known as hyperthermia. This can be achieved using magnetic NPs, which are categorized into magnetic alloy NPs (MANPs) and magnetic metal oxide NPs (MMONPs). These NPs are able to accumulate in the tumor tissue and produce heat under a magnetic field (Peiravi et al., 2022).

In a recent study, an implantable hybrid magnetic nanofibers device was designed to be used for magnetic hyperthermia and pH-dependent anticancer drug release in the tumor (Sasikala et al., 2016). For this aim, Fe_3_O_4_ NPs were mixed with a PLGA solution to prepare the electrospinning solution. After electrospinning, a shell of polydopamine was grown through a simple immersion on the surface of as-prepared magnetic nanofibers. The polydopamine-based shell with numerous catechol moieties on the surface of nanofibers was able to attach to bortezomib (BTZ), an anticancer agent. According to the results, they found this smart system highly beneficial owing to its higher therapeutic efficacy and low toxicity towards normal cells and also the potential of magnetic NPs for repeated hyperthermia application and controlled drug release in the tumor tissue (Sasikala et al., 2016).

In a similar study, Radmansouri et al. (2018) developed DOX-loaded chitosan/cobalt ferrite/titanium oxide nanofibers to achieve pH-dependent DOX release along with hyperthermia to treat melanoma. Hybrid nanofibers were prepared by co-blending chitosan with cobalt ferrite NPs, titanium oxide NPs, and DOX. According to their drug-release study, the fast release of DOX from nanofibers was seen at low pH by alternating the magnetic field. Also, cytotoxicity results exhibited considerable cell death in the combination of chemotherapy and hyperthermia.

#### 6.1.4 Cardiovascular drugs

Cardiovascular diseases, such as stroke, heart failure, and hypertensive heart disease, are the leading cause of death globally.

Different therapeutic approaches have been developed for the treatment of cardiovascular diseases. For example, arterial stents are the most used approach for coronary heart disease. Arterial stents are used to keep the artery open and prevent its obstruction to maintain continuous blood flow (McGinty, 2014). In order to enhance the efficiency of this approach, Kersani et al. (2020a) fabricated hybrid electrospun nanofibers loaded with simvastatin to cover self-expandable NiTiNOL stents. Nanofibers were produced by electrospinning a polymeric mixture containing chitosan and β-cyclodextrin (CD), forming a polyelectrolyte complex for loading simvastatin. These nanofibers showed high drug loading capacity along with excellent mechanical properties. However, more preclinical tests are necessary to investigate the biocompatibility of these implants (Kersani et al., 2020a).

Nicorandil is a vasodilator that can be administered for angina pectoris, a chest pain resulting from episodes of transient myocardial ischemia. In a recent study, this drug was loaded into electrospun nanofibers for sublingual administration to minimize mucosal ulceration (i.e., the main adverse effect of sublingual administration of nicorandil) and enhance the bioavailability of the drug (Singh et al., 2016). Polymeric nanofibers were prepared with hyaluronic acid, PVA, and vitamin B12. In this study, hyaluronic acid was used for its viscoelastic properties, which ensure the sustained release of a loaded drug with an extended retention time at the site of administration (Joshi et al., 2016) and vitamin B12 was employed due to its promising positive effects on mucosal ulceration (Kalia et al., 2016). According to histopathological results, there was no evidence of mucosal ulceration at the application site, and the preclinical safety of the as-prepared nanofibers was proved. Results showed that these biocompatible nanofibers had a high potential for sublingual administration of nicorandil and improved its low bioavailability (Singh et al., 2016).

#### 6.1.5 Ophthalmic drugs

Eyes are repeatedly washed by tears to eliminate irritants and help the immune system (Farandos et al., 2015). The usual approach for treating ocular diseases is using eye drops which are the saline solutions of drugs used directly over the eye. However, rapid turnover of the tear film, small available surface for drug absorption, and several physiological barriers lead to poor bioavailability of eye drops. Therefore, designing solid delivery systems for ocular diseases receives a great deal of attention due to their potential for improving the bioavailability of drugs resulting from reduced clearance of the solid drug delivery systems compared to liquid ones (Mishima et al., 1966; Everitt and Avorn, 1990). Grimaudo and Concheiro (2020) formulated nanofibers made of hyaluronan and polyvinylpyrrolidone (PVP) for the ocular delivery of ferulic acid (an antioxidant) and ε-polylysine (an antimicrobial peptide). According to cytocompatibility assays, the nanofibrous scaffolds showed no hemorrhage or coagulation, and there was no difference between the scaffolds and saline solution as the control. Hence, it was regarded as non-irritant. In addition, they exhibited potent antibacterial effects against *p. aeruginosa* and *S. aureus*. Considering the fast erosion of scaffolds, drug release was fast, taking place within 20 min. Thus, this designed nanofibrous scaffold was applicable for short-term medication (Grimaudo and Concheiro, 2020).

### 6.2 Tissue engineering

Tissue engineering is an emerging field in biomedicine that applies the principles of biomedical sciences and engineering to develop biological alternatives to restore, preserve or ameliorate tissue functions. In order to create an efficient tissue, the designed structures should simulate ECM, enable oxygen and nutrient circulation as well as eliminate metabolic waste during the process of tissue regeneration. A lot of effort has recently been put into providing 3D scaffolds for tissue engineering. Among these, electrospinning is one of the most promising methods. In recent years, many nanofiber-based scaffolds have been designed for tissue engineering (Rahmati et al., 2021). This section presents the application of nanofiber-based scaffolds for different tissue regeneration.

#### 6.2.1 Bone tissue engineering

Bone tissue engineering refers to designing scaffolds for delivering therapeutic agents and cells to the damaged tissue to provoke bone regeneration (Qu et al., 2019). In the design of scaffolds for bone tissue engineering, there are some considerations, including 1) the size of porosity, 2) appropriate mechanical properties and adjustable biodegradation kinetics, 3) interconnected open porosity for growth factors, 4) biocompatibility of materials used for scaffolds, and 5) sterile and suitable environment for cell growth (Qu et al., 2019).

In other words, designed scaffolds for bone tissue engineering should not only provide structure and mechanical robustness for the tissue but also mimic the ECM and stimulate bone repair. Electrospun nanofibers can provide many of the above criteria for bone tissue engineering. Different natural or synthetic polymers can be applied for designing bone tissues, such as chitosan, alginate, PCL, and polyglycolic acid (PGA).

For instance, Rachmiel et al. (2021) developed a composite nanofibrous scaffold by core-shell electrospinning of PCL and hyaluronic acid containing a short self-assembling peptide. Hyaluronic acid is one of the major ECM components responsible for modulating many biological processes. It has been stated that incorporating hyaluronic acid can enhance cell migration and proliferation in the prepared scaffolds. However, hyaluronic acid has low mechanical strength and a significant amount of free charge carriers (Shin et al., 2016). Therefore, electrospinning of pure hyaluronic acid would be challenging, and it needs to be combined with other polymers to fabricate electrospun nanofiber scaffolds. In order to modify the mechanical properties of hyaluronic acid, a low-molecular-weight hydrogelator, Fmoc-phenylalanine-arginine-glycine-aspartic acid (FmocFRGD), was employed in addition to utilizing PCL. Short synthetic peptides containing the RGD sequence can efficiently mimic ECM, promoting bone growth and accelerating cellular differentiation into osteoblasts. These favorable properties of RGD sequence can be potentiated by incorporating them into natural polymers such as hyaluronic acid (Alipour et al., 2020). In this study, the prepared scaffolds showed similar morphology to ECM and could facilitate osteogenesis (Rachmiel et al., 2021).

**FIGURE 4:**
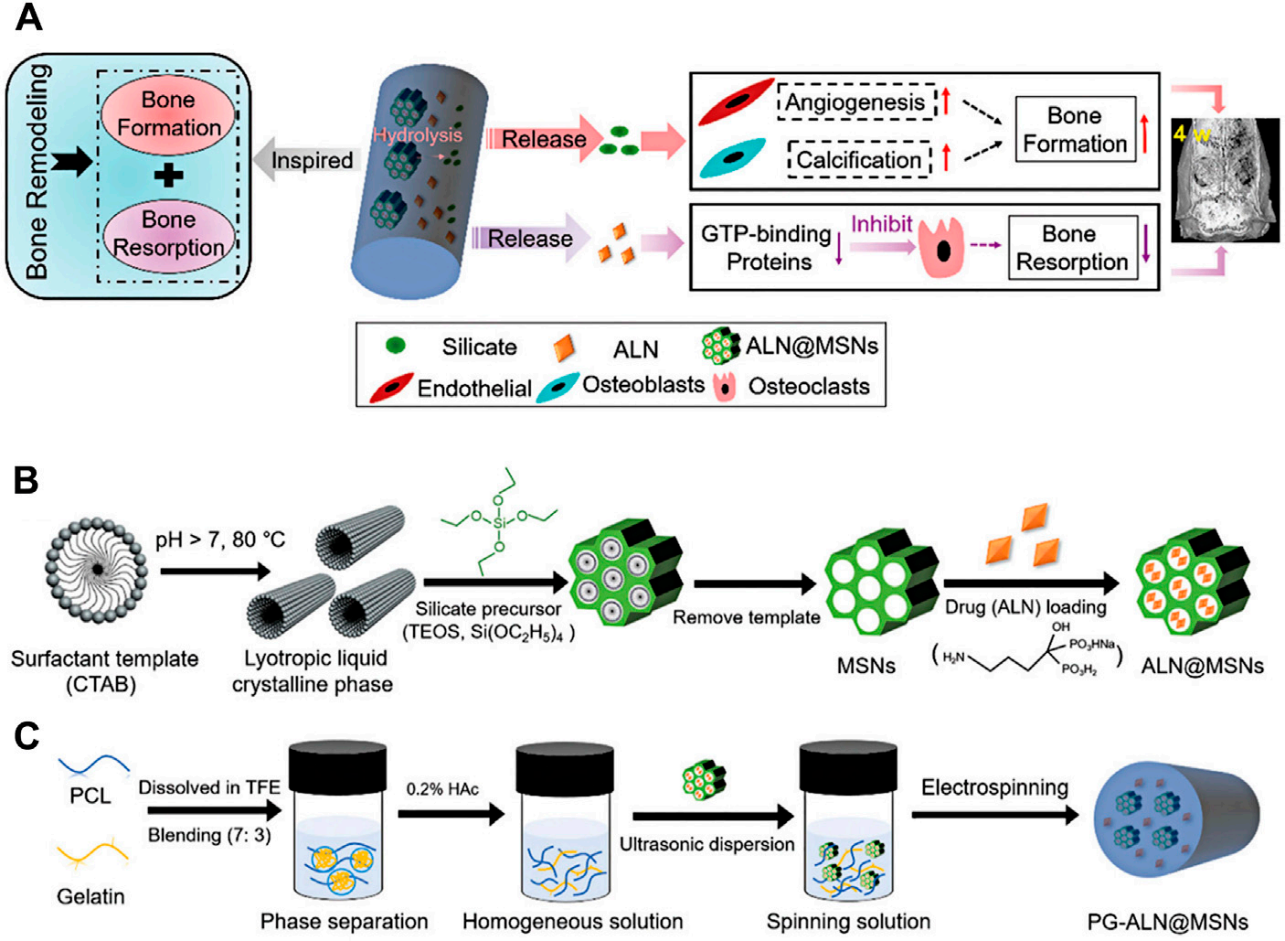
Schematic illustration of PG-ALN@MSN fabrication process and effects of prepared nanofibers on bone repair. The mechanism of action of PG-ALN@MSN for improving the bone healing process **(A)**. Preparation of ALN@MSNs **(B)**. Electrospinning of PG-ALN@MSN nanofibers **(C)**. Adapted with permission from (Wang et al., 2018b). Copyright 2018 Royal Society of Chemistry.

Wang et al. (2018b) developed a novel bioactive scaffold for regulating bone remodeling and promoting bone regeneration using mesoporous silica nanoparticles (MSNs) embedded into electrospun PCL/gelatin nanofibers. Based on the synergism effects of silicate and alendronate (ALN) − promoting bone formation with silicate and inhibiting the bone-resorbing process by ALN −, ALN was loaded into MSNs. The scaffold was successfully fabricated by co-electrospinning of acetic acid-mediated PCL/gelatin homogeneous polymeric mixture containing well-dispersed MSNs loaded with ALN. The release profile of the designed scaffolds showed that the ALN@MSN-loaded nanofibers achieved the dual release of ALN and silicate (Figure 4). In addition, *in vivo* study exhibited that the healing time in test groups was three times faster than in the control group. It seems that as-prepared electrospun nanofibers have a high potential for clinical use (Wang et al., 2018b). Table 4 summarizes recent studies on hybrid electrospun nanofibers applied in bone tissue engineering.

**TABLE 4:** An overview of electrospun hybrid nanofiber for bone tissue engineering.

Polymer	Study	Cells	Electrospinning method	Results	Ref
**(nHA/PLA/gelatin) combining BMP-2**	*In vitro*	BMSCs	Blending	✓Prepared 3D scaffolds exhibited favorable biocompatibility and osteoinductivity	Ye et al. (2019)
	*In vivo*				
**nHA/PVA/graphene oxide/COll 1**	*In vitro*	Human keratinocyte cell line (HaCaT)	Blending	✓Improved biocompatibility and mechanical properties	Rethinam et al. (2019)
**PLA/PCL**	*In vitro*	hMSCs	Blending	✓High mechanical properties	Yao et al. (2017)
	*In vivo*			✓Improved *in vitro* bioactivity	
				✓Promotion of osteogenic differentiation	
				✓Acceleration of new bone formation in a critical-sized cranial bone defect mouse model	
**SF/PLCL**	*In vitro*	Human adipose-derived stem cells (hASCs)	Blending	✓Increase of cell proliferation and osteogenic differentiation *in vitro*	Wang et al. (2016)
	*In vivo*			✓Promoted new bone formation *in vivo*	
**PCL/Zein/MoS**_**2**_	*In vitro*	Pre-osteoblasts (MC3T3-E1) cell lines	Blending	✓Improved *in vitro* biocompatibility	Awasthi et al. (2020)
				✓Increase of cell attachment, proliferation and differentiation	
**PCL/HA combining with FmocFRGD**	*In vitro*	MC3T3-E1	Coaxial electrospinning	✓Similar morphology with ECM	Rachmiel et al. (2021)
	*In vivo*			✓Acceleration of osteogenesis	
**PCL/PVA-BMP2**	*In vitro*	BMSCs	Coaxial electrospinning	✓Increase of cell proliferation, attachment and osteogenic differentiation	Huang et al. (2020)
	*In vivo*				
**PCL/gelatin/ALN@MSN-**	*In vitro*	MC3T3-e1 cells	Blending	✓Accelerating healing process	Wang et al. (2018b)
	*In vivo*			✓Enhanced cell adhesion, spreading, proliferation, differentiation, mineralization	
**PLA/Coll/HA**	*In vitro*	Mouse osteoblasts MC3T3-E1	Blending	✓High stability for up to 80 days in physiological simulated conditions	Zhou et al. (2017)
**PCL/gelatin/HA/niobium pentoxide**	*In vitro*		Blending	✓Improved cell proliferation and differentiation	Marins et al. (2019)
**PCL/PLA**	*In vitro*	hMSCs	thermally induced (nanofiber) self-agglomeration (TISA) method	✓Satisfying density, porosity, water absorption capacity, and mechanical properties	Xu et al. (2018)
				✓Improved bioactivity, and cell viability	
				✓Increasing the PLA improved stiffness/bioactivity of scaffold	
**PCL/CS/Mg/HA**	*In vitro*	Osteoblast precursor cell line (MG63)	Blending	✓Improved Cell viability and proliferation	Sedghi et al. (2020)
				✓Promoting bone mineralization	
**SPI/PEO**	*In vitro*	rBMSC	Blending	✓Improving bone formation in the presence of rBMSC	Lee et al. (2019a)
**CS/HA/G** *p*	*In vitro*	7F2 osteoblast-like cells	Blending	✓Increasing the osteoinductive bioactivity	Frohbergh et al. (2012)
				✓Suitable for non-weight bearing bone tissue engineering	
**PCL/gelatin combining with SDF-1α**	*In vitro*	BMSCs	Blending	✓Inducing the BMSCs recruitment after adding SDF-1α to the fibers	Ji et al. (2013)
	*In vivo*				
**PCL/Aloe vera/SF/HA**	*In vitro*	ASCs	Blending	✓Improved bone regeneration	Shanmugavel et al. (2013)
**PEOT/PBT/calcium phosphate**	*In vitro*	hMSCs	Blending	✓Stimulating osteogenesis	Nandakumar et al. (2010)
	*In vivo*				
**PCL/PLGA**	*In vitro*	rBMSCs	Blending	✓Enhanced cell infiltration and cartilage matrix deposition	Yang et al. (2013)
	*In vivo*			✓Improved new bone formation after 8 weeks of *in vivo* implantation	
**PCL/gelatin/GelMA nanofiber microspheres (NMs)**	*In vitro*	BMSCs HUVECs	Blending, electrospray and surface conjugation techniques	✓Enhanced osteogenesis	John et al. (2019)
				✓Enhanced angiogenesis	

nHA: nano-Hydroxyapatite, PLA: polylactic acid, BMP-2: Bone morphogenetic protein 2, PVA: polyvinyl alcohol, Coll: Collagen, PCL: polycaprolactone, HA: hyaluronic acid, FmocFRGD: Fmoc-phenylalanine-arginine-glycine-aspartic acid, SF: silk fibroin, PLCL: Poly(L-lactide-co-ε-caprolactone), ALN: alendronate, MSN: mesoporous silica nanoparticle, CS: chitosan, PEO: polyethylene oxide, SPI: soya protein isolate, GP: genipin, PEOT: Poly(ethylene oxide therephthalate), PBT: Poly(butylene terephthalate), PLGA: Poly(lactide-co-glycolide), MSC: mesenchymal stem cell, BMSC: bone marrow mesenchymal stem cell, hMSC: human mesenchymal stem cell, hASC: human adipose-derived stem cell*,* rBMC: rabbit bone marrow derived mesenchymal stem cells, SDF: stromal derived factor, HUVEC: human umbilical vein endothelial cells, GeMA: gelatin methacrylate.

#### 6.2.2 Cartilage tissue engineering

Articular cartilage defects are considered one of the challenging issues in medicine. It can progress to osteoarthritis associated with high healthcare costs and negatively influence patients’ lives. Conventional therapeutic strategies are ineffective and mainly focus on relieving the pain or delaying tissue degradation. To this end, tissue engineering opens the door to treating articular cartilage defects. To date, various scaffolds with variable degrees of efficiency have been developed for such disorders. Among them, nanofibrous scaffolds have a high potential in cartilage engineering.

Chondroitin sulfate (CHS) is a major component of cartilage that plays a critical role in regulating many chondrocytes signaling pathways (Tang et al., 2019). Moreover, CHS can act as an antioxidant and immune regulating agent (Zou et al., 2020). Irani et al. (2020) fabricated electrospun hybrid nanofibers composed of PVA/gelatin/CHS with different concentrations of CHS (10%, 15%, 20%). They exhibited that a medium concentration of CHS (15%) had better biocompatibility than other test groups. In addition, they showed that after 21 days, cultured mesenchymal stem cells (MSCs) on the prepared scaffold could produce type II collagen. However, the mechanical properties of the scaffolds were not investigated in this study (Irani et al., 2020).

3D scaffolds are more desirable for tissue engineering than 2D scaffolds (He et al., 2020). Hence, Li et al. (2019) developed 3D spongy scaffolds by adding a decellularized cartilage matrix (DCECM) into gelatin/PCL electrospun nanofibers by utilizing the homogenization and freeze-drying method for cartilage tissue engineering. The resulting fabricated scaffold had good mechanical properties, stability, and biocompatibility. In addition, the DCEM components in the scaffold could enhance the proliferation rate of chondrocytes along with the secretion of collagen and glycosaminoglycan, leading to the early maturation of cartilage lacunae (Li et al., 2019). Table 5 provides an overview of major hybrid nanofibrous scaffolds used for cartilage tissue engineering applications.

**TABLE 5:** An overview of electrospun hybrid nanofiber for cartilage tissue engineering.

Polymer	study	cell	Electrospinning method	Highlights	Ref
**GAS/PCL**	*In vitro*	ACS	Coaxial electrospinning	✓Improving cell proliferation and growth by adding GAS.	Chen et al. (2020b)
**PCL/gelatin/DCEM**	*In vitro*	ACS	Blending	✓Increasing proliferation rate of chondrocytes and early maturation of cartilage lacunae	Li et al. (2019)
	*In vivo*				
**PVA/gelatin/CHS**	*In vitro*	MSCs	Blending	✓A medium concentration of CHS (15%) had better biocompatibility than other test groups	Irani et al. (2020)
**PCL/gelatin/PEG**	*I vitro*	CPS	Blending	✓Improving cell attachment and proliferation by adding gelatin	Semitela et al. (2020)
				✓Increasing pore size and interconnectivity of fibers by adding gelatin	
**PGS/PCL/KGN**	*Invitro*	hBMSC	Coaxial electrospinning	✓Improving cell proliferation and chondrogenic differentiation by adding KGN into nanofibers	Silva et al. (2020)
**Collagen/PLCL**	*In vitro*	Chondrocytes	Blending	✓Stimulating cartilage-like tissue	He et al. (2013)
	*In vivo*			✓Significantly increasing Young’s modulus of the scaffolds by PLCL	
**CS/PVA/CaCO3**	*In vitro*	ATDC5 (Chondrogenic cell line)	Blending	✓Enhancing the diameter of fibers and Young’s modulus by increasing the concentration of CaCO3	Sambudi et al. (2015)
**Gelatin/PCL**	*In vitro*	Chondrocytes	Blending	✓High stability	Xue et al. (2013)
	*In vivo*			✓Good elasticity and mechanical strength	
**Gelatin/PLGA**	*In vitro*	Primary rabbit chondrocytes	Blending	✓Good elasticity and water-induced shape memory	Chen et al. (2019a)
	*In vivo*			✓Satisfactory cartilage regeneration *in vivo*	
**PCL/PLGA**	*In vitro*	hBMSCs	Blending	✓Aligned nanofibrous scaffold with higher PLGA ratio could significantly enhance hBMMSC proliferation and differentiation to chondrocytes	Zamanlui et al. (2018)
**Gelatin/GAG**	*In vitro*	hBMSCs	Blending	✓Scaffold comprising 15% GAG is better results for chondrogenesis	Honarpardaz et al. (2019)
**Silk/Cellulose**	*In vitro*	hMSCs	Blending	✓High biocompatibility	Begum et al. (2018)
				✓Its inherent capacity to direct chondrogenic stem cell differentiation	
**PCL/PEO**	*In vitro*	Rabbit synovial stem cell	Blending	✓High potential for repairing the meniscal hoop structure	Shimomura et al. (2019)
	*In vivo*			✓Preventing the progression to cartilage degeneration	
**PLA/gelatin**	*In vivo*	-	Blending	✓Accelerating cartilage repair *via* the PI3K/AKT signaling pathway	Yu et al. (2018)
**Gelatin/PCL**	*In vitro*	Swine auricular chondrocytes	blending	✓Improving subcutaneous cartilage regeneration in an autologous swine model	Zheng et al. (2021)
**CS/PEO**	*In vitro*	human articular chondrocytes (hACs)	Blending	✓High stability for up to 7 months	Ching et al. (2021)
				✓High biocompatibility	
**PLGA/gelatin**	*In vivo*	-	Blending, freeze-drying, and 3D printing	✓Good elasticity and water-induced shape memory	Chen et al. (2019a)
				✓Enhanced cartilage regeneration	
**PLGA/gelatin/CDM**	*In vivo*	-	Blending, 3D printing	✓Enhanced cartilage regeneration	Chen et al. (2020c)

PCL: polycaprolactone, GAS: glucosamine sulfate, DCEM: decellularized extracellular matrix, PVA: polyvinyl alcohol, CHS: chondroitin sulfate, PEG: polyethylene glycol, PGS: poly(glycerol sebacate), KGN: kartogenin, GAGs: Glycosaminoglycans*,* CS: chitosan, PLCL: Poly(L-lactide-co-ε-caprolactone), PLGA: Poly(lactide-co-glycolide), PEO: polyethylene oxide, PLA: polylactic acid, ACS: articular chondrocytes, CPS: cartilage progenitor cell line, MSC: mesenchymal stem cell, hBMSC: human bone marrow mesenchymal stem cell, hASC: human adipose-derived stem cell*,* hAC: human articular chondrocytes. CDM: cartilage decellularized matrix.

#### 6.2.3 Vascular tissue engineering

Nanofibers can also be applied for vascular tissue engineering and renovating blood vessels for clinical applications. The high porosity of nanofibers facilitates nutrient and gaseous transports leading to angiogenesis and vascular regeneration (Fraisl et al., 2009). In designing vascular scaffolds, some critical parameters should be taken into consideration by researchers, including the tensile stiffness, elasticity, and compressibility of a blood vessel (Thottappillil and Nair, 2015). Due to the high versatility of electrospun nanofibers, they can be used for developing tubular scaffolds for vascular tissue engineering. For instance, Yazdanpanah et al. (2015) fabricated nanofibrous scaffolds with a blend of PLA and gelatin. Considering the tensile test results, nanofibrous PLA/gelatin scaffolds improved mechanical strength and estimated burst pressure compared to layered PLA/gelatin and gelatin scaffolds (Yazdanpanah et al., 2015). In another study, Yang et al. (2020b) used electrospinning to fabricate nanofibrous scaffolds based on PCL and fibrin. They showed that a 20:80 PCL/fibrin graft in rats had good hemocompatibility and cytocompatibility along with satisfying mechanical properties and stability for 9 months.

Interestingly, the PCL/fibrin scaffold exhibited a better response to vasoconstrictors and vasodilators as the native artery compared to the pure PCL graft. In addition, similar to the native artery, a PCL/fibrin scaffold could increase cell infiltration, tissue regeneration, and deposition of ECM proteins, including collagen, elastin, and GAGs. They suggested PCL/fibrin scaffold as a promising tissue engineering material for vascular grafts (Yang et al., 2020b). Table 6 summarizes the major hybrid nanofibrous scaffolds utilized in vascular tissue engineering applications.

**TABLE 6:** An overview of electrospun hybrid nanofiber for vascular tissue engineering.

Polymer	Study	Cell	Electrospinning Method	Highlights	Ref
**PLA/gelatin**	*In vitro*	-	Co-electrospinning	✓Improved mechanical strength and estimated burst pressure	Yazdanpanah et al. (2015)
**PCL/Fibrin**	*In vitro*	MSCs	Blending	✓Increased cell proliferation, infiltration and tissue regeneration	Yang et al. (2020b)
	*In vivo*			✓Satisfying mechanical properties and stability for 9 months in rats	
**PU/PEG**	*In vitro*	HUVECs	Blending	✓The mechanical properties of the scaffolds are close to those of human and pig arteries	(Wang et al., 2012b)
				✓Improved hemocompatibility by adding PEG.	
				✓Improved cell attachment and proliferation	
**Elastin/** *p* **U/Coll**	*In vitro*	SMCs	Blending	✓The scaffolds attain viscoelastic properties in the presence of elastin	Wong et al. (2013)
				✓Improved cell proliferation in the presence of collagen	
				✓Increase of cell growth and proliferation	
**PCL/CollI**	*In vitro*	SMCs	Blending	✓Providing a mature smooth muscle layer that expressed robust cell-to-cell junction	Ahn et al. (2015)
				✓Increasing the growth and proliferation of cells	
**PET/PU**	*In vitro*	Fibroblast	Blending	✓Enhanced mechanical properties in the range of native vessels	Hasan et al. (2015)
				✓Non-toxi	
**PCL/PLA/Coll**	*In vitro*	HUVECs	Multi-layer (sequential electrospinning)	✓Promptly inhibition of the first thrombus formation	Haghjooy Javanmard et al. (2016)
				✓Acceleration of endothelialization	
				✓Increasing vascular regeneration	
**PCL/Coll**	*In vitro*	L929, ECs SMCs	Coaxial electrospinning	✓Good biocompatibility and cell affinity properties	Duan et al. (2016)
**PLA/PCL combining with HDFs, HUVEC**	*In vitro*	HUVECs	Blending	✓Improving the collagen remodeling and biomechanical properties up to day 14	Lee et al. (2015)
	*In vivo*			✓Increasing the cell growth and proliferation	
**PVA/gelatin/PCL**	*In vitro*	HUVECs	Co-electrospinning	✓High porosity	Tan et al. (2016)
	*In vivo*			✓Improved cell growth and proliferation	
**PCL/PU**	*In vitro*	Human endothelial cell line (EA.hy926)	Template-assisted electrospinning	✓High cell viability	Abdal-Hay et al. (2018)
**PCL/PVA/alginate**	*In vitro*	HUVECs	Template-assisted electrospinning	✓Mechanical and structural properties resembled native vessels	Liu et al. (2016)
				✓Enhanced cell integration, adhesion, and growth	

PLA: polylactic acid, PCL: polycaprolactone, PEG: poly ethylene glycol, PU: polyurethan, Coll: Collagen, PVA: polyvinyl alcohol, PET: polyethylene terephthalate, MSC: mesenchymal stem cell, HDF: human dermal fibroblast, HUVEC: human umbilical vein endothelial cells, SMC: vascular smooth muscle cell, EC: Endothelial cell.

#### 6.2.4 Cardiac tissue engineering

Myocardial infarction is one of the most prevalent disorders, leading to cardiomyocyte death due to an interruption in nutrient and oxygen transport in the myocardium. Following the infraction, the myocardium usually loses its regeneration ability. Therefore, implanting tissue-engineered myocardium into the damaged tissue is the easiest approach to compensate for tissue damage resulting from the infarction. The main challenge in myocardial tissue regeneration is providing an environment similar to native cardiac tissue (Weinberger et al., 2017; Lodrini and Goumans, 2021). Electrospun nanofiber-based scaffolds have been explored for cardiac tissue engineering. They are able to simulate myocardium ECM structure to increase cell adhesion, viability, and tissue regeneration. High conductivity and elasticity to mimic cardiac function are necessary for developing cardiac tissue scaffolds (Rahmati et al., 2021). Conductive nanofibrous scaffolds are highly desirable for cardiomyocytes-based bioactuators. However, few studies have worked on such scaffolds.

Developing conductive nanofibrous scaffolds by electrospinning would be beneficial for cardiomyocytes-based bioactuators, but such scaffolds have rarely been reported. The work performed by Rahmati et al. (2021) presented a conductive nanofibrous sheet with tunable conductivity based on polylactide and polyaniline (PANI) *via* electrospinning. These conductive nanofibrous sheets had the ability to enhance cardiomyocytes’ maturation and spontaneous beating. In addition, they could form cardiomyocyte-based 3D bioactuators with tubular and folding shapes, which indicated their great potential in cardiac tissue engineering and bioactuators’ applications (Rahmati et al., 2021).

Wang et al. (2017) designed conductive nanofibers based on PANI, an electroconductive polymer, and PLA composition as a cardiac tissue scaffold. The prepared PANI/PLA hybrid nanofibers displayed suitable biocompatibility and increased cellular interaction and spontaneous beating of primary cardiomyocytes. Furthermore, a satisfying amount of cardiomyocytes-based 3D bioactuators with tubular and folding shapes were formed.

Dippold et al. (2016) formulated elastic nanofibers composed of poly (glycerol sebacate) (PGS) and zein for cardiac engineering. Results exhibited that adding zein to PGS could augment the mechanical properties of PGS, and the fibers had good biocompatibility as well as stability for cardiac tissue engineering. In a more recent study, Kumar et al. (2020) prepared an aligned PCL/gelatin coaxial nanofiber patch *via* the electrospinning method. The cells on cardiac patches had synchronous contraction with a fast response to therapeutic agents. Therefore, they suggested the designed patches for *in vitro* drug screening in cardiotoxicity studies (Kumar et al., 2020). Table 7 summarizes the major hybrid nanofibrous scaffolds utilized in vascular tissue engineering applications.

**TABLE 7:** An overview of electrospun hybrid nanofiber for cardiac tissue engineering.

Polymer	Study	Cell	Electrospinning method	Highlights	Ref
**PCL/gelatin**	*In vitro*	hiPSC-CMs	Blending	✓Providing functional syncytium and synchronous calcium transients	Kumar et al. (2020)
				✓Improved response to cardiac drugs	
**PCL/gelatin**	*In vitro*	Rabbit cardiomyocytes	Blending	✓Providing anisotropic wetting and mechanical properties by the scaffolds	Kai et al. (2011)
				✓Improving cell attachment	
**PCL/PANi**	*In vitro*	(H9C2) Cardiomyoblasts	Blending	✓Suitable biocompatibility	Wang et al. (2017)
				✓Increased cellular interaction and spontaneous beating of primary cardiomyocytes	
**PLCL/POC**	*In vitro*	Cardiac myoblast	Blending	✓Increasing the cell growth and proliferation after 2 and 8 days by increasing the concentration of POC.	Prabhakaran et al. (2012)
**PLCL/SF/Aloe Vera**	*In vitro*	cardiomyocytes	Blending	✓Suitable for MI repair	Bhaarathy et al. (2014)
				✓Improved cardiac cell proliferation	
				✓The scaffolds had better cardiac expression proteins myosin and connexin after 9 days cell culture	
**PLGA/PCU**	*In vitro*	(H9C2) cardiac myoblasts	Blending	✓Improved cell growth and proliferation in anisotropic textile-templated scaffolds	Şenel Ayaz et al. (2014)
				✓Simillar mechanical properties to a human heart	
**poly (glycerol sebacate) (PGS)/zein**	*In vitro*	N/A	Blending	✓High stability over a 28-day time period by just a small drop in pH after 28 days	Dippold et al. (2016)
**PGS/PCL**	*In vitro*	(C2C12) myoblasts neonatal rat cardiomyocytes	Blending	✓Improved cell attachment over the first 8 h after seeding and aligning after 24 h	Tallawi et al. (2016)
				✓Scaffolds could direct cell responses	
**Alginate/gelatin/hyaluronic acid**	*In vitro*	Human iPSC-derived ventricular cardiomyocytes (hiPS-CM)	Wet electrospinning	✓Improved cell adhesion, migration, proliferation, and maturation	Vicini et al. (2018)
**PLGA/PCU**	*In vitro*	H9C2	Template-assisted electrospinning	✓Supported cell adhesion and proliferation	Şenel Ayaz et al. (2014)
				✓guided anisotropic organization of cardiac-like tissue	
				✓prolonged spontaneous synchronous contractility	

PCL: polycaprolactone, PANi: Poly aniline, PLCL: Poly(L-lactide-co-ε-caprolactone), SF: silk fiber, POC: Poly(1,8-octanediol-*co*-citrate), PCU: Thermoplastic polycarbonate-urethane, PLGA: Poly(lactide-co-glycolide), PGS: Poly(glycerol sebacate), hiPSC-CMs: human induced pluripotent stem cell-derived cardiomyocytes (hiPSC-CMs).

#### 6.2.5 Nerve tissue engineering

Peripheral nerve injury is one of the most common disorders which lead to devastating consequences with loss of motor and sensory function and poor quality of life. Currently, conventional treatments focus on nerve autografting to restore damaged nerves. Despite all the advances in neurology, this process has limitations, such as low efficiency and mismatching between the damaged and donor nerves. In addition, although peripheral nerves can regenerate to some extent, the results are not satisfying for serious injuries. Therefore, there is a need to find a novel approach instead of nerve autografting (Behtaj et al., 2022).

Nerve guidance conduits (NGCs) can be a promising strategy as an alternative to nerve autografts for improving functional outcomes. NGCs are tubular biostructures used to bridge nerve injury sites. Thus, they act as a guide and protective microenvironment for the regeneration and restoration of function in the target area (Behtaj et al., 2022). Designed NGS should mimic ECM to promote NGS interaction with nerves to induce neural repairment. Electrospun nanofibrous scaffolds have a high potential to replicate the native fibrous ECM and create an appropriate environment guiding nerve regeneration. In order to enhance the efficiency of nerve regeneration, the topological structure, bioelectricity, surface characteristics, permeability, degradation rate, and mechanical properties of scaffolds should be optimized (Behtaj et al., 2022).

For instance, Tian et al. (2016) fabricated a surface-modified, electrically conductive, aligned nanofibrous scaffold composed of PLA and polypyrrole (Ppy) for nerve regeneration. The effects of prepared electrospun nanofibers on neuronal differentiation using PC12 cells were assessed. In order to increase cell attachment to the nanofibers, they provided a hydrophilic surface with a Poly-ornithine coating. They showed that conductively aligned nanofibers based on PLA and Ppy guided PC12 cells’growth along the fiber direction and were advantageous for neurite outgrowth. Indeed, aligned fibers could promote cell proliferation, differentiation, and neurite outgrowth more than random fibers. Moreover, external electrical stimulation (40 mV) improved neurite outgrowth (Tian et al., 2016). Table 8 presents a summary of electrospun nanofibers used for nerve regeneration.

**TABLE 8:** An overview of electrospun hybrid nanofiber for nerve regeneration.

Polymer	Study	Cell	Electrospinning method	Highlights	Ref
**PLGA/PEG**	*In vitro*	NSCs	Blending	✓iNSCs were shown to survive, with the ability to self-renew and undergo neural differentiation into neurons and glial cells within the 3D scaffolds *in vivo*	Liu et al. (2015)
	*In vivo*			✓The iNSC-seeded scaffolds restored the continuity of the spinal cord and reduced cavity formation	
**PLA/(Ppy)**	*In vitro*	PC12	Blending	✓Aligned fibers could promote cell proliferation, differentiation, and neurite outgrowth more than random fibers	Tian et al. (2016)
				✓External electrical stimulation (40 mV) improved the neurite outgrowth	
**PVA/Keratin**	*In vitro*	Glial cell	Blending	✓High potential as nerve repair alternative with increase in cell viability and proliferation	Mahanta et al. (2014)
**PCL/gelatin**	*In vitro*	C17.2	Blending	✓High stability at 70:30 proportion	Ghasemi-Mobarakeh et al. (2008)
				✓Improved cell differentiation and proliferation	
				✓The cell direction is parallel to the direction of fibers	
*p* **(LACL)/CollI/Coll III**	*In vitro*	C17.2	Blending	✓Improving cell proliferation on aligned nanofibers	Kijeńska et al. (2012)
**PLA/PANi**	*In vitro*	NSCs	Blending	✓Protracted neurite outgrowth compared to the cells grown on non-stimulated scaffolds	Prabhakaran et al. (2011)
**PLCL/Coll**	*In vitro*	MSCs	Blending	✓Improving cell differentiation and proliferation using neuronal inducing factors such as b-mercaptoethanol, epidermal growth factor, nerve growth factor and brain derived growth factor in DMEM/F12 media	Prabhakaran et al. (2009)
**PLCL/Laminin PLCL**	*In vitro*	Rat Schwann	Coaxial electrospinning	✓Improving cell proliferation on laminin containing core–shell PLCL scaffolds	Kijeńska et al. (2014)
**PHBV/Coll**	*In vitro*	PC12	-	✓Improved cell proliferation	Prabhakaran et al. (2013)
				✓Aligned nanofibers provide contact guidance to direct the cells along the direction of fibers	
**PCL/gelatin**	*In vitro*	PC12	Blending	✓Stimulating the essential biological pathways related to nerve regeneration including cell adhesion, proliferation, the neurite outgrowth and differentiation	Alvarez-Perez et al. (2010)
**PPy/SF**	*In vitro*	Rat primary Schwann cells and mouse fibroblast cells (L929)	Multilayered electrospinning and 3D printing	✓Enhanced cell adhesion, differentiation, and proliferation	Zhao et al. (2018)

PLGA: Poly(lactide-co-glycolide), PEG: polyethylene glycol, PLA: polylactic acid, Ppy: polypyrrole, PVA: polyvinyl alcohol, PCL: polycaprolactone, PLCL: Poly (L-lactic acid)-co-poly(ε-caprolactone), Coll: collagen, PANi: Polyaniline, PLCL: Poly(L-lactide-co-ε-caprolactone), PHBV: Poly(3-hydroxybutyrate-co-3-hydroxyvalerate), NSC: neural stem cell, MSC: mesenchymal stem cell, SF: silk fibroin.

### 6.3 Wound healing

Wound healing is a complex process with different stages, including hemostasis, inflammation, proliferation, and remodeling. One of the critical steps in wound management is wound dressing to shield the wound from external risk factors and accelerate the healing process (Chen et al., 2020a; El Ayadi et al., 2020). Characteristics of an ideal wound dressing include: 1) absorbs excess exudate, 2) prevents infection, 3) keeps the moisture of the wound site, 4) ease of gas exchange, 5) is biocompatible and degradable, 6) does not stick to the wound, and easy to remove, 7) induces angiogenesis and tissue remodeling (Das et al., 2019; Fahimirad and Ajalloueian, 2019; Kanikireddy et al., 2020). Currently, different wound dressings exist in the market (e.g., hydrogels, foams, films, and nanofibers). Nanofiber membranes have received much attention among wound dressings due to their unique feature, high porosity, small pores, and large surface area. This structural feature of nanofibers can prevent pathogens’ growth in a wound’s environment and ensure gas and liquid molecules’ exchange. Electrospun nanofibers show high potential as wound dressings (Liu et al., 2021b). Table 9 offers an overview of electrospun hybrid nanofibers applied for wound dressing.

**TABLE 9:** An overview of electrospun hybrid nanofiber for wound dressing.

Polymer	Active ingredient	Study	Wound Model	Electrospinning method	Application	Highlights	Ref
**PCL/Fibrinogen**	ADSCs	*In vitro*	Excisional	Bi-layer	Wound dressing	✓Promoting re-epithelialization, angiogenesis, and collagen remodeling	Mirzaei-Parsa et al. (2019)
		*In vivo*	Wound				
			Model				
**PVA/PVP/glycerol**	*Enterococcus* mundtii QAUEM2808	*In vitro*	A second-degree contact burn	Blending	Burn wound healing	✓Accelerating epithelialization, collagen deposition, and hair follicle formation	Khan et al. (2019)
		*In vivo*				✓ Inhibiting harmful bacteria	
						✓Providing interference benefits	
**HA/COll**	PDGF and VEGF EGF and bFGF	*In vitro*	Diabetic model	Blending	Chronic wound healing	✓Providing sustained release of growth factors over 1 month	Lai et al. (2014)
		*In vivo*				✓Elevated collagen deposition and angiogenesis	
						✓Accelerated wound closure rate	
**PELA/PEG**	plasmid bFGF (pbFGF)	*In vitro*	Diabetic model	Emulsion electrospinning	Diabetic foot ulcer healing	✓Higher wound recovery rate	Yang et al. (2012)
		*In vivo*				✓Improved vascularization	
						✓Enhanced collagen deposition and maturation	
						✓Complete re-epithelialization and formation of skin appendages	
**CS/PEO**	Bromelain	*In vitro*	Induced burn wounds	Blending	Bun wound healing	✓Accelerating wound healing process	Bayat et al. (2019)
		*In vivo*					
**Gelatin/CA**	Berberine	*In vitro*	Diabetic model	Blending	Diabetic foot ulcer healing	✓Antibacterial activity	Samadian et al. (2020)
		*In vivo*					
**SA/PEO**	Lavender oil	*In vitro*	Induced burn wounds	Emulsion electrospinning	Burn wound healing	✓Improved antibacterial and anti-inflammatory activity	Hajiali et al. (2016)
		*In vivo*					
**PCL/CS**	Quercetin/rutin	*In vitro*	Induced burn wounds	Blending	Burn wound dressing	✓High antibacterial and antioxidant effects	Zhou et al. (2021)
		*In vivo*					
**CS/SF**	Astragaloside IV	*In vitro*	Acute trauma model	Blending	Wound healing	✓Potentiated wound recovery and angiogenesis	Zhang et al. (2019a)
		*In vivo*				✓Preventing scar complications	
**Gelatin/CA/PVP**	Gentamycin	*In vitro*	-	Bi-layer	Wound dressing	✓Proper thermal stability, wettability characteristics	López-Calderón et al. (2020)
						✓Antibacterial activity	
**Coll/EC/PLA**	Silver sulfadiazine	*In vitro*	-	Blending	Wound dressing	✓Antibacterial activity against *Bacillus* and *E. Coli*	Ahmadian et al. (2020)
						✓Promoted cell proliferation and adhesion	
**Collagen/Zein/PCL**	n-ZnO, aloevera	*In vitro*	-	Blending	Wound healing	✓Good cell compatibility	Ghorbani et al. (2020)
**CS/PCL**	Lidocaine hydrochloride, mupirocin	*In vitro*	Excisional	Dual	Wound dressing	✓Improved the wound healing process	Yang et al. (2020c)
		*In vivo*	Wound			✓Complete re-epithelialization as well as collagen deposition	
			Model			✓Promoting hemostasis	
						✓High antibacterial activity	
						✓Efficient drug release	
**CS/PEO/CNC**	*Acacia* extract	*In vitro*	-	Blending	Wound dressing	✓A continuous release of natural acacia extract for 24 h	Ribeiro et al. (2021)
**SF/PLGA**	Artemisinin	*Invitro*	Excisional	Blending		✓High anti-inflammatory properties without cytotoxicity	Peng et al. (2021)
		*In vivo*	Wound				
			Model				
**SF/PCL/PVA**	Curcumin	*In vitro*	Diabetic mice	Blending	Healing potential in diabetic wound	✓Accelerated wound healing in diabetic mice	Agarwal et al. (2021)
		*In vivo*					
**Alginate/PVA/CS**	Dexpanthenol	*In vitro*	-	Coaxial electrospinning	Wound dressing	✓Good biocompatibility	Najafiasl et al. (2020)
						✓Improved cell attachment	
**Alginate/PVA**	Cardamom extract	*In vitro*	-	Blending	Wound dressing	✓Good biocompatibility and antibacterial properties	Najafi et al. (2021)
**PVP/EC**	Ciprofloxacin, AgNP	*In vitro*	-	Side-by-side	Wound dressing	✓suitable bactericidal activity	Yang et al. (2020a)
**PVP/PLA/PEO/Coll**	Cefazolin	*In vitro*	Excisional	Coaxial electrospinning	Wound dressing	✓Efficient antibacterial activity	Hajikhani et al. (2021)
		*In vivo*	Wound				
			Model				
**PCL/CS**	Aloe vera	*In vitro*	-	Blending	Wound dressing	✓Satisfying antibacterial properties and biocompatibility	Yin and Xu, (2020)
**PCL/CS**	Curcumin	*In vitro*	Infected model	Blending	Wound dressing	✓Proper antibacterial, anti-oxidant and wound healing capabilities	Fahimirad et al. (2021)
		*In vivo*					
**PCL/Gelatin**	Oregano oil	*In vitro*	-	Blending	Wound dressing	✓Good biocompatibility	El Fawal et al. (2021)
						✓Appropriate antibacterial activity	
**PCL/Gelatin**	Clove essential oil	*In vitro*	-	Blending	Wound dressing	✓Good antibacterial activity	Unalan et al. (2019)
**PVA/CS/Starch**	-	*In vitro*	-	Blending	Wound dressing	✓Suitable tensile strength and elongation	Adeli et al. (2019)
						✓Good biocompatibility and antibacterial activity	
**PVA/CS**	-	*In vitro*	Excisional	Blending	Wound dressing	✓Satisfying physical and chemical properties	Wang et al. (2020b)
		*In vivo*	Wound			✓Good biocompatibility and antibacterial properties	
			Model				
**PEO/CS**	Vancomycin	*In vitro*	Excisional	Blending	Wound dressing	✓Antibacterial effects against *S. Aureus*	Kalalinia et al. (2021)
		*In vivo*	Wound				
			Model				
**PEO/CS**	Teicoplanin	*In vitro*	Excisional	Dual electrospinning	Wound dressing	✓Improved wound healing process	Amiri et al. (2020)
		*In vivo*	Wound				
			Model				
						
**CS/PVA**	-	*In vitro*	Excisional	Blending and gas-foaming post-processing	Wound dressing	✓Improved wound healing process	Zhang et al. (2019b)
		*In vivo*	Wound			✓Reduced scar formation	
			Model				

PCL: polycaprolactone, PVA: polyvinyl alcohol, PVP: polyvinylpyrrolidone, HA: hyaluronic acid, Coll, Collagen, PDGF: platelet derived growth factor, VEGF: vascular endothelial growth factor, EGF: epidermal growth factor, bFGF: basic fibroblast growth factor, PEG: polyethylene glycol, PELA: Poly (ethylene glycol-co-lactide), CS: chitosan, PEO: polyethylene oxide, CA: cellulose acetate, SA: sodium alginate, SF: silk fibroin, EC: ethyl cellulose, Coll: Collagen, PLGA: Poly(lactide-co-glycolide), CNC: cellulose nanocrystals.

#### 6.3.1 Types of wounds

Wounds refer to skin deformities or tissue discontinuities caused by physical or thermal injury or underlying ailments. Generally, wounds are divided into acute and chronic wounds. Acute wounds are usually caused by surface burns, chemical, and mechanical injuries, etc. Acute wounds can go through the normal process of wound healing. On the other hand, chronic wounds cannot go through this process and may be open for more than 1 month (Tottoli et al., 2020; Smet et al., 2021). Chronic wounds are commonly caused by particular diseases like diabetes. It is noteworthy that chronic wounds are susceptible to infection and inflammation, disturbing the process of wound healing. Chronic wounds are an important medical issue, putting a heavy burden on health care systems (Homaeigohar and Boccaccini, 2020; Smet et al., 2021).

#### 6.3.2 Electrospun nanofiber for wound healing

##### 6.3.2.1 *Cell delivery*

Full-thickness skin wounds that may result from different injuries can lead to many functional problems and strongly affect the quality of life. In these kinds of wounds, the skin cannot regenerate spontaneously due to the severity of damage to skin tissue. The standard treatment in these cases is thin split-thickness skin autografts. In this method, scar formation and donor site availability are the major hurdles. Where skin autografting is not possible, allografts or porcine xenografts are used, which are highly likely to reject the graft. Dermal cell or stem cell transplantation *via* electrospun dressings is a promising strategy for treating severe wounds (Gizaw et al., 2019; Behere and Ingavle, 2022). Different synthetic and natural polymers can be used to fabricate nanofiber-based scaffolds for skin tissue regeneration. In view of low cell attachment, proliferation, or infiltration in using only synthetic polymers, copolymerizations and blending with other hydrophilic polymers, especially combining with the skin tissue components − such as collagen, hyaluronic acid, and fibronectin—is highly recommended (Behere and Ingavle, 2022). For instance, in a recent study, Mirzaei-Parsa et al. (2019) developed nanofiber-based scaffolds for adipose-derived stem cells (ADSCs) as a wound dressing by electrospinning of PCL/fibrinogen. They reported that ADSCs seeded on nanofibers showed the best results for promoting re-epithelialization, angiogenesis, and collagen remodeling compared to the control and other tested groups (Mirzaei-Parsa et al., 2019).

In order to prevent infection in the damaged area, particularly for severe and chronic wounds, maintaining a skin-balanced microbiome is helpful. Probiotic strains have shown considerable healing properties for burn wounds. The potential of using probiotics for accelerating wound healing led to the development of probiotic (*Enterococcus* mundtii QAUEM2808)-functionalized PVA/PVP/glycerol electrospun scaffolds for wound dressing (Khan et al., 2019). They exhibited that obtained scaffolds could enhance probiotic survival with no disturbance in the function of probiotics. The scaffolds were degraded over time in a simulated wound fluid which is crucial for activating probiotic strains to inhibit Gram-positive and Gram-negative pathogens. Additionally, the scaffolds could accelerate re-epithelialization, collagen deposition, and hair follicle formation. Furthermore, they prevented bacterial infection and provided interference benefits in a second-degree contact burn on the dorsum of male BALB/c mice (Khan et al., 2019).

##### 6.3.2.2 *Growth factors*

Due to the important role of growth factors in skin regeneration, using growth factors in wound dressings can accelerate the healing process. Lai et al. (2014) designed collagen and hyaluronic acid inter-stacking nanofibrous scaffolds containing four angiogenic growth factors, including vascular endothelial growth factor (VEGF), platelet-derived growth factor (PDGF), basic fibroblast growth factor (bFGF), and epidermal growth factor (EGF). It has been revealed that the delivery of EGF and bFGF in the early stage can improve epithelialization and vasculature sprouting. In contrast, the release of PDGF and VEGF in the late stage can stimulate blood vessel maturation (Lai et al., 2014).

Thus, for earlier release of EGF and bFGF, they were freely added into the polymeric matrix, whereas PDGF and VEGF were loaded into gelatin NPs to provide extended release of these growth factors in the damaged site. The mechanical properties of prepared scaffolds were the same as native skin and could provide sustained release of growth factors over 1 month. The scaffolds indicated elevated collagen deposition and angiogenesis on streptozotocin (STZ) induced diabetic rats and accelerated wound closure rate. Altogether, the composite nanofibrous electrospun scaffolds with a stage-wise release pattern of multiple angiogenic factors can be a promising candidate for chronic wound healing (Lai et al., 2014).

In order to provide prolonged release of growth factors in the damaged area, transgene expression of growth factors in dermal cells has been developed (Laiva et al., 2018). Nanofibers have the potential to be employed for the delivery of DNA. There are different strategies to incorporate plasmid DNA into fibrous scaffolds. Among them, core-sheath electrospun structures and DNA condensation techniques are the most efficient strategies that result in the desired DNA release profile (Afsharian and Rahimnejad, 2021). For instance, plasmid bFGF (pbFGF) polyplex with poly (ethylene imine) were incorporated into poly (dl-lactide)-poly-(ethylene glycol) (PELA)/PEG nanofibers with core-sheath structures by emulsion electrospinning for skin wound healing in diabetic rats (Yang et al., 2012). The sustained release of pbFGF could considerably improve wound recovery rate, vascularization, collagen deposition, and maturation, leading to complete re-epithelialization and formation of skin appendages.

##### 6.3.2.3 *Drugs*

In order to accelerate the process of wound healing and regeneration, nanofibers can be loaded with different drugs such as antibiotics, pain relievers, etc. For instance, Varshosaz et al. (2020) prepared a wound dressing membrane based on hybrid nanofibers using modified polybutylene adipate-co-terephthalate and gelatin, loaded with doxycycline, by using the double electrospinning technique. In these nanofibrous structures, polybutylene adipate-co-terephthalate was used to enhance the mechanical properties, and gelatin was applied to provide a water-soluble part inside the scaffold to control the release and achieve better ECM mimicry. Moreover, to improve cell adhesion, prepared electrospun nanofibers were modified by RGD, a peptide with a critical role in cell adhesion to the ECM. Also, the presence of doxycycline in nanofibers could prevent bacterial infection in the wound environment, particularly strains of *S. aureus* and *p. aeruginosa*. The nanofibers showed considerable positive effects on the wound healing process within 3 days after initiation of the treatment. However, the used polymers in the composition of nanofibers did not show noticeable improvement in the scaffold’s mechanical properties (Varshosaz et al., 2020).

In another study, pH-responsive coaxial nanofibers were developed to co-deliver two drugs—lidocaine hydrochloride used for pain relief and curcumin as an anti-inflammatory agent (Guo et al., 2020). In this study, the composition of chitosan/PEO was used as a shell part for loading lidocaine, and PCL containing curcumin was placed at the core. Additionally, sodium bicarbonate was used within the core layer to provide a pH-responsive release of curcumin. The pH-responsive release of drugs in this system was achieved by employing chitosan and sodium bicarbonate. Under an acidic environment, chitosan was protonated, and lidocaine was released. Also, sodium bicarbonate generated CO_2_ by reacting with hydrogen ions, resulting in many holes in nanofiber structures and curcumin release. This novel design led to an immediate release of lidocaine to decrease pain, and when the inflammatory phase started—leading to a more acidic wound environment—the release of curcumin was intensified. It is noteworthy that curcumin could exert antibacterial effects against *E. coli* and *S. aureus* (Guo et al., 2020)*.*

Burns are another major skin damage that may be life-threatening or influence the quality of life. Usually, skin damages caused by burning are more susceptible to bacterial infection due to loss of skin integrity. In a recent study, Hadisi et al. (2020) developed novel core-shell nanofibers with hyaluronic acid and silk protein. Hyaluronic acid was selected among various natural polymers because of its high potential in accelerating wound healing by modulating inflammatory responses, angiogenesis, and cell migration. However, due to its insufficient mechanical properties and fast degradation, it had to be combined with another polymer with high mechanical resistance. Therefore, silk protein was chosen to solve the mentioned drawbacks of hyaluronic acid. In addition, zinc oxide was added to the blend of hyaluronic and silk protein owing to its antibacterial effects. Prepared hybrid nanofibers showed noticeable healing effects and antibacterial properties against *E. coli* and *S. aureus* in a scratch assay *in vitro* and *in vivo* (Hadisi et al., 2020).

##### 6.3.2.4 *Herbal compounds*

Bromelain, a mixture of proteolytic enzymes derived from pineapple tissues, is a natural debriding agent which can remove fibrin clots in the burned area. Furthermore, bromelain can improve and accelerate the healing process in the damaged area. In a recent study done by Bayat et al. (2019), PEO/chitosan nanofibers were loaded with bromelain by the electrospinning method. These nanofibers showed considerable healing effects and decreased burned lesions in the animal model. Complete regeneration of skin was achieved when applying these fibrous nanostructures (Bayat et al., 2019).

Zhang et al. (2019a) prepared a nanofibrous electrospun based on silk fibroin (SF) and gelatin polymers loaded with Astragaloside IV (AS-IV) for promoting wound healing and relieving scars *in vivo* in an acute trauma model. AS-IV is a natural active component with antioxidant, immunostimulant, anti-inflammatory, and angiogenic effects. Results showed that the SF/gelatin/AS-IV nanofibers could potentiate wound recovery and angiogenesis. Additionally, they prevented scar complications. Quercetin is a flavonoid derived from rutin with remarkable antioxidant activity found in fruits and vegetables. Quercetin and rutin have a high potential to be used for wound healing, considering their favorable characteristics, including antioxidant, anti-inflammatory, and antibacterial activities and pain management.

Zhou et al. (2021) prepared a membrane of electrospun fibers composed of PCL, chitosan oligosaccharides, and quercetin/rutin to heal burned skin. Developed nanofibers exhibited high antibacterial and antioxidant effects in the wound environment. Generally, this study showed PCL/chitosan/Quercetin/Rutin nanofibers’ potential for developing burn dressings.

Lawsone (Law) is a quinone derived from the leaves of the henna plant. In a recent study, lawsone was incorporated into PCL/gelatin nanofiber by the coaxial electrospinning technique and tested for wound healing activity. This compound was successfully entrapped into the core polymer. As a result of wound healing activity, PCL/gelatin/Law mats of 0.5% and 1% increased the expression of transforming growth factor-beta (*TGF-β1*) and collagen type 1(*COL1*) genes as well as rat wound re-epithelialization *in vivo* (Adeli-Sardou et al., 2019).

Lavender oil is an essential oil with various pharmacological effects, including antibacterial, antifungal, carminative (smooth muscle relaxant), sedative, and wound healing properties for burns and insect bites (Cavanagh and Wilkinson, 2002). Hajiali et al. (2016) prepared electrospun nanofibers with a blend of sodium alginate (SA) and PEO embedded with lavender oil. The SA-PEO/lavender oil fibers could effectively inhibit bacterial colonies and also inflammation, resulting in a considerable decrease in interleukin 6 (IL-6) (66%) and IL-8 (49%) concentrations. The SA-PEO/lavender oil nanofibers in the animal model could also moderate cytokine production and inflammation and improve wound recovery without leaving marks or spots on the skin (Hajiali et al., 2016).

### 6.4 Biosensors

**FIGURE 5:**
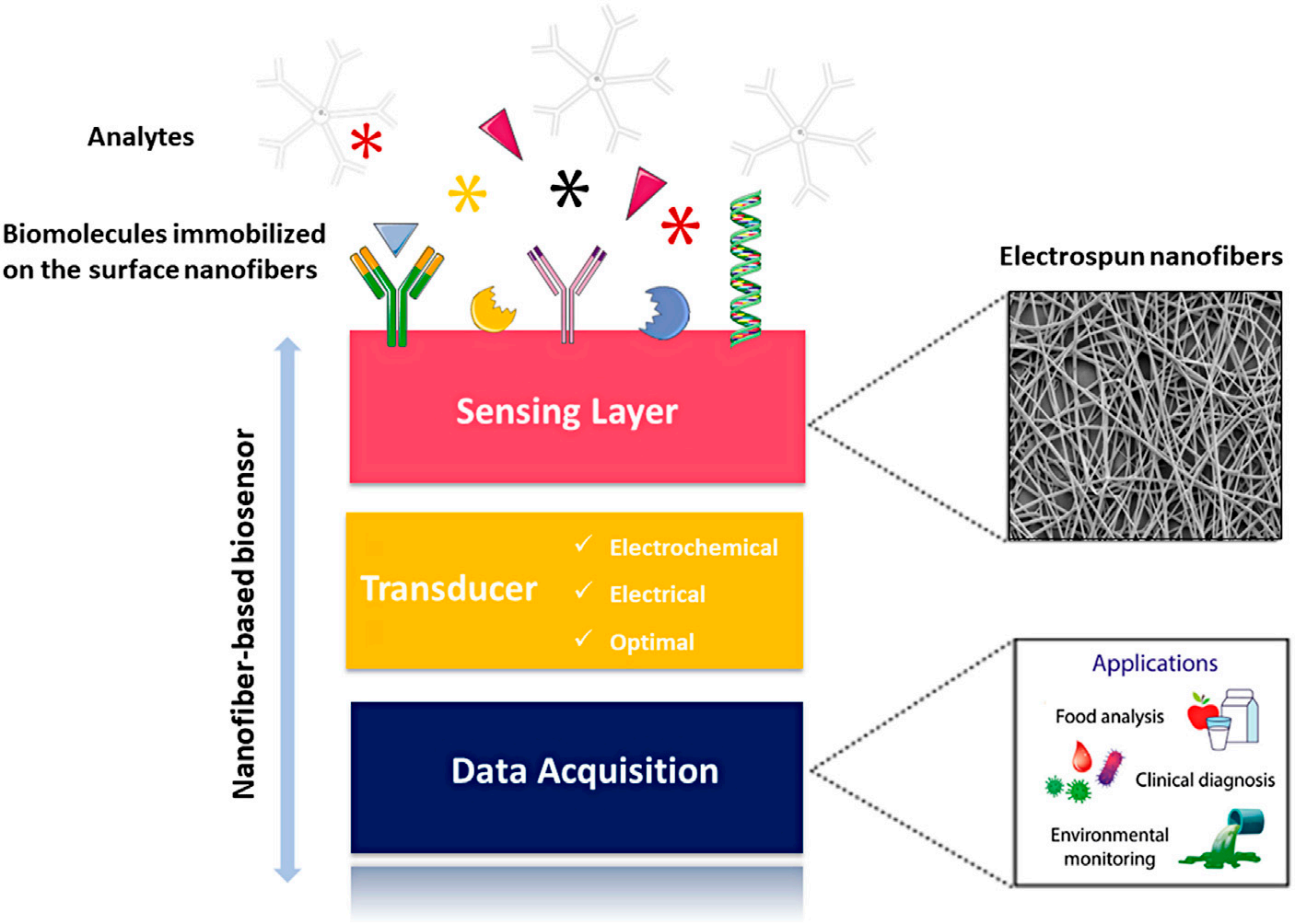
Schematic illustration of a nanofiber-based biosensor device.

Biosensors are analytical devices that play an important role in both research and the market—owing to their potential to detect various substrates for clinical diagnostics (Li et al., 2021b), food analysis (Ali et al., 2020), and environmental monitoring (Hara and Singh, 2021). Biosensors have some advantages compared to conventional sensors, such as portability, selectivity, and sensitivity. In addition, they can offer a fast, facile, and cost-effective assay of analytes (Dincer et al., 2019). Biosensors consist of three main parts: 1) a biorecognition element for sensing the analyte, 2) a transducer for converting the biological response into a quantitative signal, and 3) a signal capture for measuring signals derived from biological interactions (Figure 5). Indeed, biosensors are based on biological interactions between recognizing biomolecules and target analytes and converting them to measurable signals. The functionality and efficacy of biosensors are associated with the materials used to immobilize bio-receptors (Cavalcante et al., 2021). In this process, the biomolecules should fix on the transducer’s surface, and their functionality should be maintained during the assay (Scouten et al., 1995). In recent years, nanomaterials have received increasing attention for engineering electrode surfaces into appropriate immobilization matrices (Guan et al., 2008). For example, high surface area and ease of surface functionalization in nanofibers make them an attractive candidate for the development of biosensors. The large surface area of nanofibers can increase biosensors’ sensitivity by enhancing the possibilities of interaction with analytes (Mercante et al., 2017).

Moreover, the high porosity of nanofibers leads to low mass transport resistance, thereby elevating analyte diffusion through the sensing layer (Zhang et al., 2017a). According to aimed application, the mechanical properties, hydrophilicity, and stability of nanofibers should be modified by adjusting electrospinning parameters and materials used. In order to enhance the interaction site for the target analyte and biosensors’ sensitivity, the composition of nanofibers should be modulated. In this regard, various synthetic and natural polymers have been used for designing nanofiber-based biosensors. Along with synthetic and natural polymers, conductive polymers including poly (3,4-ethylene dioxythiophene) (PEDOT) (Yang et al., 2014), poly (pyrrole) (PPy) (Hazarika and Kumar, 2017), and PANI (Scagion et al., 2016) can also be combined with other macromolecules to provide the obtained nanofibers with electrical, electrochemical and electromechanical properties. Nanostructures, such as inorganic NPs (Zhu et al., 2013), graphene quantum dots (Zhang et al., 2015), CNTs (Li et al., 2014), and reduced graphene oxide (rGO) (Pavinatto et al., 2018), can be added to polymeric matrices to improve nanofiber-based biosensors’ transducer performance or increase their capability for sensing analytes by modulating mechanical, optical, electrical, and thermal characteristics of nanofibers (Mercante et al., 2017). However, optimizing the material composition in these cases is challenging due to the high tendency of NPs for aggregation, which can disturb the sensing function of biosensors (Mercante et al., 2017).

## 7 Conclusion and future perspectives

In recent years, electrospinning technology has attracted increasing attention for fabricating micro or nanofibers. Electrospun nanofibers have some outstanding characteristics, including high surface-to-volume ratios, high porosity, low cost, easy fabrication and surface functionalization, and adjustable fiber morphology, making them attractive for biomedical use. A wide range of polymers with different sources can be applied for hybrid nanofiber fabrication. Generally, polymers are divided into natural and synthetic polymers. Biopolymers are non-toxic with higher biocompatibility and biodegradability compared to synthetic polymers.

Additionally, they may possess unique biological activities such as antibacterial, antifungal, and anticancer effects. Despite all their benefits, biopolymers have poor mechanical properties compared to synthetic polymers. Hence, biopolymers and synthetic polymers can be combined and make hybrid nanofibers to benefit from the advantages of both polymers. There are several techniques to prepare electrospun hybrid nanofibers with different structures. Furthermore, functional agents such as drugs, biomolecules, and NPs can be incorporated into a polymeric matrix to fabricate multifunctional hybrid nanofibers.

The main and basic setup to fabricate hybrid nanofibers include: 1) blend electrospinning, 2) coaxial or triaxial electrospinning, 3) emulsion electrospinning, and 4) side-by-side electrospinning. The future will bring more advanced and complex multi-fluid electrospinning technologies for producing nanofibers with unique collective properties.

Electrospun nanofibers are ideal candidates for various biomedical and healthcare applications, especially the antimicrobial treatment of bacteria-related biofilms, orthopedic implant-related infections, tissue scaffolds, drug delivery, wound dressings, and biosensors. However, some challenges should be considered to improve the functionality of nanofibers. For instance: 1) the pore size of nanofibrous scaffolds is too small, making cell infiltration difficult. 2) The mechanical strength of electrospun nanofiber-based scaffolds is usually not enough for hard tissue regeneration. 3) Controlling *in vivo* degradation of nanofibers is a complicated process 4) Industrial fabrication of electrospun hybrid nanofibers remains challenging. It is noteworthy that although nanofibers have shown promising results in preclinical studies, many clinical studies are still needed to ensure their clinical applications.

In the future, it is necessary to improve the function of electrospun nanofibers and develop new functional nanofibers to provide more technical support for biomedical use. As a result of the trend and prospects, nanofiber-miniaturized systems are increasingly used for next-generation biomedicine platforms in clinical and point-of-care studies. In addition, the combination of 3D printing and computational simulation with electrospinning is the potential trend. Ultimately, we believe all these will be achieved in the future with the development of science and technology.
